# Combining MEK and SRC inhibitors for treatment of colorectal cancer demonstrate increased efficacy in vitro but not in vivo

**DOI:** 10.1371/journal.pone.0281063

**Published:** 2023-03-23

**Authors:** Fan Fan, Susmita Ghosh, Reid Powell, Jason Roszik, Yongsun Park, Mary Sobieski, Alexey Sorokin, Clifford Stephan, Scott Kopetz, Lee M. Ellis, Rajat Bhattacharya

**Affiliations:** 1 Surgical Oncology, The University of Texas M D Anderson Cancer Center, Houston, Texas, United States of America; 2 Center for Translational Cancer Research, Institute of Biosciences and Technology, Texas A&M University, College Station, Texas, United States of America; 3 Melanoma Medical Oncology, The University of Texas M D Anderson Cancer Center, Houston, Texas, United States of America; 4 Gastrointestinal Medical Oncology, The University of Texas M D Anderson Cancer Center, Houston, Texas, United States of America; 5 Molecular and Cellular Oncology, The University of Texas M D Anderson Cancer Center, Houston, Texas, United States of America; Albert Einstein Cancer Center: Albert Einstein Medical Center, UNITED STATES

## Abstract

Metastatic colorectal cancer (mCRC) is the second leading cause of cancer deaths in the United States. More than 50% of patients with mCRC harbor mutations of the oncogenic driver RAS (KRAS or NRAS). Because directly targeting most mutations of RAS is technically challenging, researchers have concentrated on targeting MEK, a downstream mediator of RAS. However, targeting MEK as single-agent therapy is ineffective in patients with mCRC. We hypothesize that combining a MEK inhibitor with other agents can enhance the efficacy of MEK targeting in mCRC. Unbiased high-throughput screening (HTS) was performed to identify drugs that enhance the efficacy of MEK inhibitors. HTS was performed with KRAS-mutated CRC cells using the MEK inhibitor trametinib as a “backbone” and two “clinically ready” compound libraries approved by the U.S. Food and Drug Administration or in clinical trials. HTS demonstrated that the combination of the SRC inhibitor dasatinib and trametinib was synergistic in CRC cells *in vitro* (MTT and colony formation assays). Analysis of markers for cell proliferation and apoptosis using fluorescence-activated cell sorting, reverse-phase protein array, or Western blotting demonstrated decreased cell proliferation and increased cell death when targeting both SRC and MEK as compared to single agents in multiple CRC cell lines. However, combining dasatinib and trametinib *in vivo* at doses in mice equivalent to doses used in humans failed to significantly enhance the antitumor activity of trametinib when compared to that of trametinib alone. These results underscore the importance of performing careful preclinical *in vivo* validation studies using clinically relevant doses as a prerequisite for translating in vitro findings to the clinic.

## Introduction

Colorectal cancer (CRC) is the second leading cause of cancer-related deaths in the United States, with approximately 50,000 deaths each year [1]. Although the median overall survival for patients with unresectable metastatic CRC (mCRC) has improved modestly, the response rate for current systemic combination therapies is about 50%, and most patients die of their disease within 2.5 years of diagnosis of metastasis [2]. Targeted therapies have improved survival over that with chemotherapeutics alone, but the benefits are still modest and only increase the overall survival by a few months [3]. Among the new therapeutic approaches, immunotherapies have proven to be effective in a subset of patients (4–5%) with mCRC who have tumors with mismatch repair defects [4–6]. Thus, identifying and developing innovative therapies are urgently needed for the majority (~95%) of patients with mCRC [7, 8].

CRC is a complex disease, with multiple factors playing important roles in treatment and prognosis [9–13]. Mutated RAS (KRAS or RAS) dictates the biology of the disease; about 50% of patients with mCRC have tumors harboring RAS mutations, contributing to therapy resistance and poor outcomes [1, 2, 14–18]. Thus, developing therapies to target RAS has been a logical step toward improving outcomes for a large group of patients with mCRC. However, direct targeting of most RAS mutants has been technically challenging [19]. Only recently has the KRAS G12C mutation been successfully targeted [20], and treatment with an inhibitor of KRAS G12C both alone and in combination with cetuximab produced early encouraging responses in patients with KRAS G12C-mutated CRC [21–23]. Due to historical challenges in directly targeting RAS, investigators have concentrated on targeting MEK, a downstream mediator of RAS. However, single agents targeting MEK in patients with RAS- or RAF-mutated mCRC have not been effective [24]. In clinical trials, attempts to combine PI3K/AKT inhibitors with MEK have also failed [25]. Preclinical studies demonstrating the efficacy of targeting of Bcl-xL [26] or CDK4/6 [27, 28] in combination with MEK inhibitors have led to early-phase clinical trials (NCT02079740 and NCT01037790) with outcomes pending. Recently, combining autophagy inhibitors with MEK inhibitors in treatment of CRC and pancreatic cancer has shown promise *in vitro* and is being studied further in anticipation of advancement to clinical trials [29–31]. However, more work must be done to identify successful combination therapies to improve the efficacy of MEK inhibitors, specifically in patients with RAS-mutated mCRC.

In this study, we hypothesized that MEK-targeted therapy for mCRC is most effective when combined with other agents, either chemotherapeutics or targeted therapeutics, identified by unbiased screening approaches. We performed 2D high-throughput screening (HTS) using the MEK inhibitor trametinib as a backbone and two different clinically “ready” compound libraries: 1) the National Cancer Institute oncology set V of approved compounds and 2) a custom set of clinical compounds either approved by the U.S. Food and Drug Administration or tested in clinical trials. We identified the SRC inhibitor dasatinib as being synergistic with trametinib in KRAS-mutant CRC cells in preliminary *in vitro* studies. We evaluated the efficacy of this combination therapy further both *in vitro* and *in vivo*.

## Materials and methods

### Cell culture

The human CRC cell lines HCT116 (KRAS G13D), SW620 (KRAS G12V), DLD-1 (KRAS G13D), LS174T (KRAS G12D), and SW480 (KRAS G12V) were purchased from the ATCC. The CRC cell line HCP-1 (KRAS G12D) was developed in our laboratory and described previously [32]. The CRC cells were cultured in minimum essential medium. All media were supplemented with 5% fetal bovine serum (Atlanta Biologicals) and the recommended concentrations of vitamins, nonessential amino acids, penicillin/streptomycin, sodium pyruvate, and L-glutamine (Thermo Fisher Scientific). All experiments were performed using cells within 15 passages. All cell lines were validated at The University of Texas MD Anderson Cancer Center Cytogenetics and Cell Authentication Core facility. Before plating, the cell number and viability were determined using a Cellometer (Nexcelom Bioscience) according to the manufacturer’s instructions.

### 2D High-throughput screening (HTS)

The compound library used in the primary screen to identify compounds synergistic with trametinib consists of 242 U.S. Food and Drug Administration-approved and phase 3 investigational drugs acquired from the National Cancer Institute. All drugs were diluted in DMSO on Echo certified low-dead-volume plates (Labcyte). All drugs were transferred from a low-dead-volume source plate into assay plates using an Echo 550 liquid handling platform (Labcyte). All wells containing CRC cells were treated with a fixed amount of DMSO (0.5%; v/v). For combination assays, DMSO was backfilled to a final concentration of 0.5% (v/v) following the addition of the drugs. Wells containing DMSO and media served as on-plate negative controls.

For the 2D HTS screening CRC cells were plated on 384-well plates and allowed to attach to the wells for 24 hours. The plates are then treated with drugs (alone and in combination) and incubated for 72–96 hours. After incubation, the cells are fixed with methanol and stained with DAPI, and images of them are taken for analyses. A rigor and reproducibility analysis of the controls across assay plates demonstrated this to be a highly robust assay, with an average z-prime (Z’) values ranging from 0.65–0.87 across all cell lines tested (S1 Fig).

### 2D monolayer screening

The optimal cell-seeding density for each CRC cell line was determined using a preliminary growth assay. In the growth assay, five cell-seeding densities (125, 250, 500, 1,000, and 2,000 cells/well) were sampled at 0, 24, 48, 72, and 96 hours. Sustained logarithmic growth and resolvable nuclei at the end of the experimental window were the criteria used to select initial seeding densities. Cells were plated onto optical bottom 384-well clear plates (Greiner Bio-One) using a multichannel pipette for growth assays or a Multidrop liquid dispenser (Thermo Fisher Scientific) for screening assays. All cells were grown in a 37°C incubator with high humidity and 5% CO_2_. For screening assays, cells were plated and allowed to recover overnight, treated as described in the previous section, and incubated in the presence of each drug for 72 hours. Plates were then fixed and DAPI-stained to facilitate cell counting. Briefly, media were aspirated from wells using a BioTek plate washer, and 4% paraformaldehyde was added to each well for 5 minutes at room temperature. The cells were then permeabilized with a 0.1% (v/v) solution of Triton X-100/PBS and counterstained with DAPI (Thermo Fisher Scientific). The plates were washed and kept in a 0.4% solution of paraformaldehyde/PBS. Plates were then imaged using an IN Cell Analyzer 6000 (GE Healthcare) with a Plan Apo 4x/0.20 NA objective (Nikon), which covers the entirety of each well. IN Cell Developer software (GE Healthcare) was used to automatically count nuclei, and cell counts were exported to an Excel spreadsheet (Microsoft Corporation).

### MTT and colony formation assays

CRC cells (2,000–5,000) were plated in 96-well flat-bottom plates. After 24 hours, cells were treated with trametinib, dasatinib, or both. The cells grew further for 72 hours, and their survival was measured using 3-(4,5-dimethylthiazol-2-yl)-2,5-diphenyltetrazolium bromide (MTT) reagent [33].

For colony formation assays, CRC cells were plated in six-well dishes (3,000–5,000 cells/well) for 24 hours. Different numbers of cells were used as growth rates and colony formation patterns of different CRC cells are different. Next, various drugs alone or in combination were added to the wells at different doses as required, and cells were allowed to grow for an additional 7 days. The surviving colonies were stained with 0.05% methylene blue solution and imaged, and the stained colonies were counted using ImageJ software (National Institutes of Health) [34]. Cell-bound dye was then extracted from the wells using 1% SDS, and the optical density of these extracted solutions was measured at 600 nm. Synergy of combining the drugs was determined using the Bliss additivity model from the optical density values measured from the extracted dyes.

For MTT assays HCT116 cells were treated with 1 nM trametinib, 25 nM dasatinib, and combination of the two. DLD-1 cells were treated with 50 nM trametinib and 500 nM dasatinib. HCP-1 cells were treated with 10 nM trametinib and 1000 nM dasatinib. SW620 cells were treated with 0.5 nM trametinib and 10 nM dasatinib. SW480 and LS174T cells were treated with 1 nM trametinib and 500 nM dasatinib. Drug doses were determined by initially treating each cell line with various doses of trametinib and dasatinib individually and identifying doses that result in <50% cell growth inhibition.

### Western blots

Proteins in CRC cell lysates were separated via SDS-PAGE following a standard protocol and transferred to Immobilon PVDF membranes (EMD Millipore). Membranes were blocked with 5% milk in Tris-buffered saline with 0.1% Tween 20 (TBST) for 1 hour followed by incubation with a primary antibody (diluted in blocking buffer or 3% bovine serum albumin in TBST) overnight. Membranes were then washed three times in TBST and reincubated with horseradish peroxidase-labeled secondary antibodies for 1 hour, washed three times in TBST, and exposed to autoradiography films. Signals on membranes were detected via chemiluminescence (Thermo Fisher Scientific). Antibodies against pERK1/2 (cat. #9101; RRID:AB_331646), ERK1/2 (cat. #4695; RRID:AB_390779), pAKT (cat. #9271; RRID:AB_329825), AKT (cat. #9272; RRID:AB_329827), pSRC (cat. #6943; RRID:AB_10013641), SRC (cat. #2123; RRID:AB_2106047), pFAK (cat. #3284; RRID:AB_10831810), FAK (cat. #3285; RRID:AB_2269034), pMEK (cat. #9154; RRID:AB_2138017), MEK (cat. #9126; RRID:AB_331778), PARP (cat. #9542; RRID:AB_2160739), cleaved PARP (cat. #9541; RRID:AB_331426), caspase 3 (cat. #9665; RRID:AB_2069872), and cleaved caspase 3 (cat. #9661; RRID:AB_2341188) were obtained from Cell Signaling Technology. Antibodies against vinculin (cat. #sc-25336; RRID:AB_628438) were purchased from Santa Cruz Biotechnology. All antibodies were used according to the manufacturers’ specifications.

All CRC cell lysates were prepared in RIPA buffer with protease and phosphatase inhibitors as described previously [35]. All tissue lysates were prepared by sonicating residual tumor pieces obtained at the end of in vivo experiments and suspended in RIPA buffer with protease and phosphatase inhibitors followed by centrifugation to remove cellular debris.

### Reverse Phase Protein Array (RPPA)

RPPA analyses were performed at the MD Anderson Functional Proteomics RPPA Core Facility as described previously [36]. Briefly, cell lysates were serially diluted twofold for five dilutions (from undiluted to 1:16 dilution) and arrayed on nitrocellulose-coated slides. Samples were probed with antibodies using catalyzed signal amplification and visualized in a DAB colorimetric reaction. Slides were scanned on a flatbed scanner to produce 16-bit TIFF images of the reacting spots, and spot densities were quantified using MicroVigene software (VigeneTech). Relative protein levels in each sample were determined via interpolation of dilution curves from the "standard curve" constructed by a script in the R computing language written by members of the MD Anderson Department of Bioinformatics and Computational Biology. Heat maps of proteins were generated using Cluster 3.0 software (http://bonsai.hgc.jp/~mdehoon/software/cluster/software.htm) as a hierarchical cluster using Pearson correlation and a center metric.

### Flow cytometry

HCP-1, HCT116 and SW620 cells (0.1 × 10^6^) were seeded in six-well plates. After 24 hours, trametinib, dasatinib, or both were added to the plates, and the cells were further grown for 72 hours. Cells were then washed and stained for annexin V and propidium iodide for 15 minutes at room temperature in the dark using the FITC Annexin V Apoptosis Detection Kit I according to the manufacturer’s protocol (BD Biosciences; cat. #556547; RRID:AB_2869082).

### Patient-derived xenografts

KRAS-mutated CRC patient-derived xenografts (PDXs), C1138 (KRAS G13D), and C1142 (KRAS G12V), were obtained from a repository at MD Anderson Cancer Center through a collaboration with Dr. E. Scott Kopetz. All PDXs were collected through a protocol approved by the MD Anderson Institutional Review Board (IRB).

### In vivo studies

PDXs verified to have KRAS mutations were initially grown subcutaneously in NOD *scid* gamma mice as described previously [27, 37]. PDXs that grew to ~1 cm was collected from euthanized animals and dissected into small (~2 mm) pieces. PDXs were implanted subcutaneously into flanks of anesthetized nude mice and allowed to grow to ~100–200 mm^3^. The animals were then randomized to treatment with a vehicle, trametinib, dasatinib, or both trametinib and dasatinib. Each treatment group consisted of 10 mice. The drugs were prepared as 10% PEG400 and 5% Tween 80 suspensions and administered via oral gavage (5 days on, 2 days off). Tumor sizes were measured twice every week using slide calipers by blinded observers.

For *in vivo* studies using CRC cells, HCT116, SW620, or LS174T cells (1 × 10^6^) were subcutaneously implanted into flanks of nude mice as described previously [38]. Treatments and tumor measurements were done as described above with 10 animals/ treatment group.

### Ethics statement

All in vivo experiments utilizing PDXs were performed according to NIH NCI recommendations summarized in SOP50102: PDX Implantation, Expansion and Cryopreservation (Subcutaneous). Tumor specimens were obtained from patients with mCRC under a research laboratory protocol (LAB10-0982) approved by UT MD Anderson Cancer Center Institutional Review Board (IRB), and all patients provided written informed consent for specimens to be used for research purposes including implantation in xenografts. Xenografts were established in 6–8-week-old female NSG mice. All *in vivo* studies to determine drug efficacy were performed using 6–8-week-old nude mice. Either male or female mice were used for experiments with a specific tumor/PDX. All studies were performed in accordance with accepted guidelines for housing, euthanasia and treatment, under a protocol (# 00001368-RN00) approved by UT MD Anderson Cancer Center Institutional Animal Care and Use Committee (IACUC).

### Bliss synergy analysis

High-throughput combinatorial screens to identify drug synergy were performed by testing varying stoichiometries of the anchor and probe drugs. This approach effectively minimizes the false-negative rate and maximizes the chance of detecting synergistic interactions when compared with a single-point or fixed-ratio experimental approach. To provide additional rigor and automated outlier detection, the data were fit to a 3D surface using a support vector machine-based method as described previously [39]. The theoretical additivity surface was then calculated using a Bliss independence model [40]. Drug-drug interactions (antagonistic, additive, or synergistic) were determined by comparing the empirically determined drug effect on CRC cell survival with the theoretical Bliss independence.

### Statistical analyses

All graphical representations of results and statistical analyses were done using Excel software. Two-tailed Student *t*-tests were performed to compare groups, with the results expressed as mean (± SEM) values. *P* values less than 0.05 were considered significant. For *in vitro* assays, all quantitative values had at least three replicates. For the *in vivo* studies, 8–10 tumors in each treatment group were measured. 10 mice/group were estimated to provide a sample size to achieve 80% power to detect differences in tumor growth of ~50% at a significance level of 0.05.

## Results

### HTS identified the SRC inhibitor dasatinib to be synergistic with the MEK inhibitor trametinib in KRAS-mutated CRC cell lines

**Fig 1:**
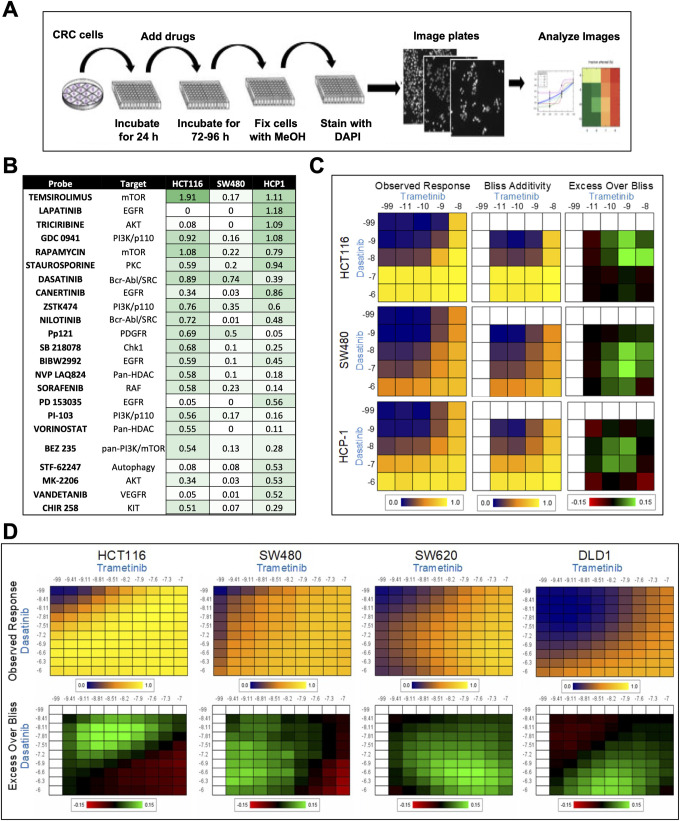
Use of HTS to identify drugs that are synergistic with trametinib. **A,** schematic of 2D HTS performed to identify drugs that are synergistic with trametinib. **B,** table of synergy score values for drugs combined with trametinib. A subjective cutoff value greater than 0.5 was considered to indicate synergy. The drugs tested (probes), their targets, and the synergy scores for the cell lines HCT116, SW480, and HCP-1 are shown. **C,** representative pairwise synergy plots for CRC cells. The left panels show the experimentally observed growth inhibitory/cytotoxic activity (observed response) of single agents and all pairwise combinations of concentrations of trametinib and dasatinib. Concentrations of trametinib (x-axis) and dasatinib (y-axis) are shown at log scale. The middle panels show the Bliss additivity surfaces subsequently calculated using the single-agent dose response. In both of these panels, blue denotes negative control-like activity, and yellow denotes strong activity. In the right panel, the difference between the experimental observed response and predicted Bliss surface is shown. Drug concentrations are shown at log scale. In this panel green denotes synergistic regions, and black denotes additive regions. **D,** results of validation studies of the combination of trametinib and dasatinib. Various CRC cell lines were grown in the presence of trametinib (x-axis) and dasatinib (y-axis) at extended concentration ranges. The top panels show the effects of various doses of single-agent and combination treatment on cell growth (observed response) with blue denoting negative control-like activity and yellow denotes strong activity. The bottom panels show the excess over Bliss as a measure of synergy at the same doses. The drug concentrations are shown at log scale. In the bottom panels green denotes synergistic regions, and black denotes additive regions.

We performed 2D HTS screening to identify drugs that are synergistic with the MEK inhibitor trametinib. We used trametinib as the base compound and performed multidose pairwise combinations using 242 clinically relevant drugs as described previously [41]. Fig 1A depicts the 2D HTS screening protocol. Initially, we subjected the KRAS mutated CRC cell lines HCT116, SW480, and HCP-1 to the 2D high-throughput combinatorial screen. From the resulting data, we calculated the cumulative excess over Bliss scores to provide a ranking of the drug synergy. This revealed multiple synergistic drug pairs, which are summarized in Fig 1B. Among these combinations, we identified classes of PI3K/mTOR, SRC, and receptor tyrosine kinase inhibitors that are broadly synergistic with trametinib in KRAS-mutant cell lines. Importantly, some of these classes were previously identified as synergistic to MEK inhibitors and with our current knowledge of cell signaling pathways, these findings were anticipated; these findings increased our confidence that this method provides relevant information [42–45].

Although multiple PI3K/mTOR inhibitors demonstrated strong synergy with trametinib in our HTS assays, previously described clinical evidence indicated that combining these two classes of drugs leads to enhanced toxicity in patients [42]. Thus, we decided to examine the efficacy of combined treatment of CRC cells with the SRC inhibitor dasatinib and trametinib (Fig 1C). We proceeded to validate these classes of drugs using extended pairwise combination analysis while also testing the generalizability of the combination of dasatinib and trametinib in additional KRAS-mutant cell lines. In these assays, we validated that dasatinib was synergistic with trametinib in four KRAS-mutated CRC cell lines (Fig 1D). The top panels in Fig 1D demonstrate that the drug combination had a strong effect on inhibition of cell proliferation, whereas the lower panels (the green areas represent excess over Bliss) indicate drug-synergy at the same doses. Collectively, these data provided a strong rationale for advancing the combination of dasatinib with trametinib into additional *in vitro* and *in vivo* model systems.

### Dual inhibition of SRC and MEK is synergistic in multiple KRAS-mutated CRC cell lines

**Fig 2:**
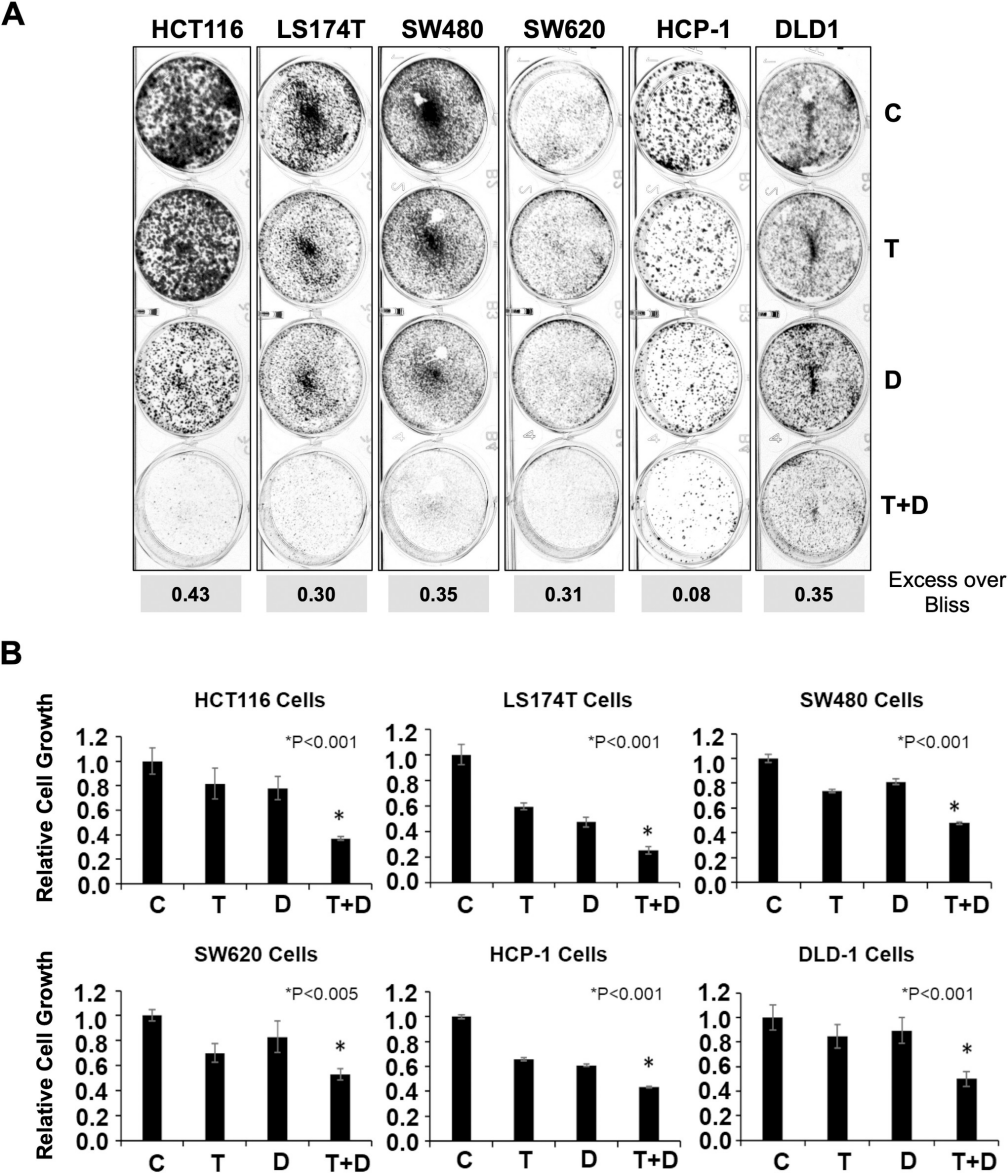
The combination of SRC and MEK inhibition is synergistic in multiple KRAS-mutated CRC cell lines. **A**, the results of colony formation assays performed to demonstrate the synergy of trametinib and dasatinib in multiple KRAS-mutated CRC cell lines. Representative images of different cell lines are shown. The numbers in the boxes below the images indicate synergy (excess over Bliss). C, control; T, trametinib; D, dasatinib; T+D, trametinib and dasatinib. **B**, the results of MTT assays performed using different KRAS-mutated CRC cell lines to further validate the enhanced efficacy of combining trametinib with dasatinib as compared with either drug alone or DMSO (control). All cells were treated for 72 hours. The data were normalized to control (taken as 1.0) and relative cell growth were plotted. All data are presented as mean (± SD) values. *P* values shown are for combination treatments when compared with trametinib. All *P* values were generated using the student *t*-test. Note: the IC_50_ was determined for each drug for every cell line using MTT assays. Cells were treated with similar to or lower than the IC_50_ of each drug to demonstrate enhanced efficacy of the drug combinations.

We determined the effect of the combination of dasatinib and trametinib on long-term cell viability using a colony formation assay. In a panel of CRC cells with KRAS mutation, treatment with this combination suppressed colony formation to a greater extent than did treatment with either drug alone (Fig 2A). We measured the extent of cell growth retardation as described in Materials and Methods and calculated the synergy of combining dasatinib and trametinib using the Bliss model of synergy (S2 Fig). The differences from Bliss indicating synergy are represented as the numbers in the boxes at the bottom of Fig 2A.

We further validated the effect of the combination of dasatinib and trametinib on cell viability using MTT assays. In each of six KRAS-mutated CRC cell lines, the combination had a greater effect on inhibition of cell survival than did either dasatinib or trametinib alone or DMSO (control) (Fig 2B).

### The combination of dasatinib and trametinib inhibits compensatory survival signaling pathways in multiple KRAS-mutated CRC cell lines

**Fig 3:**
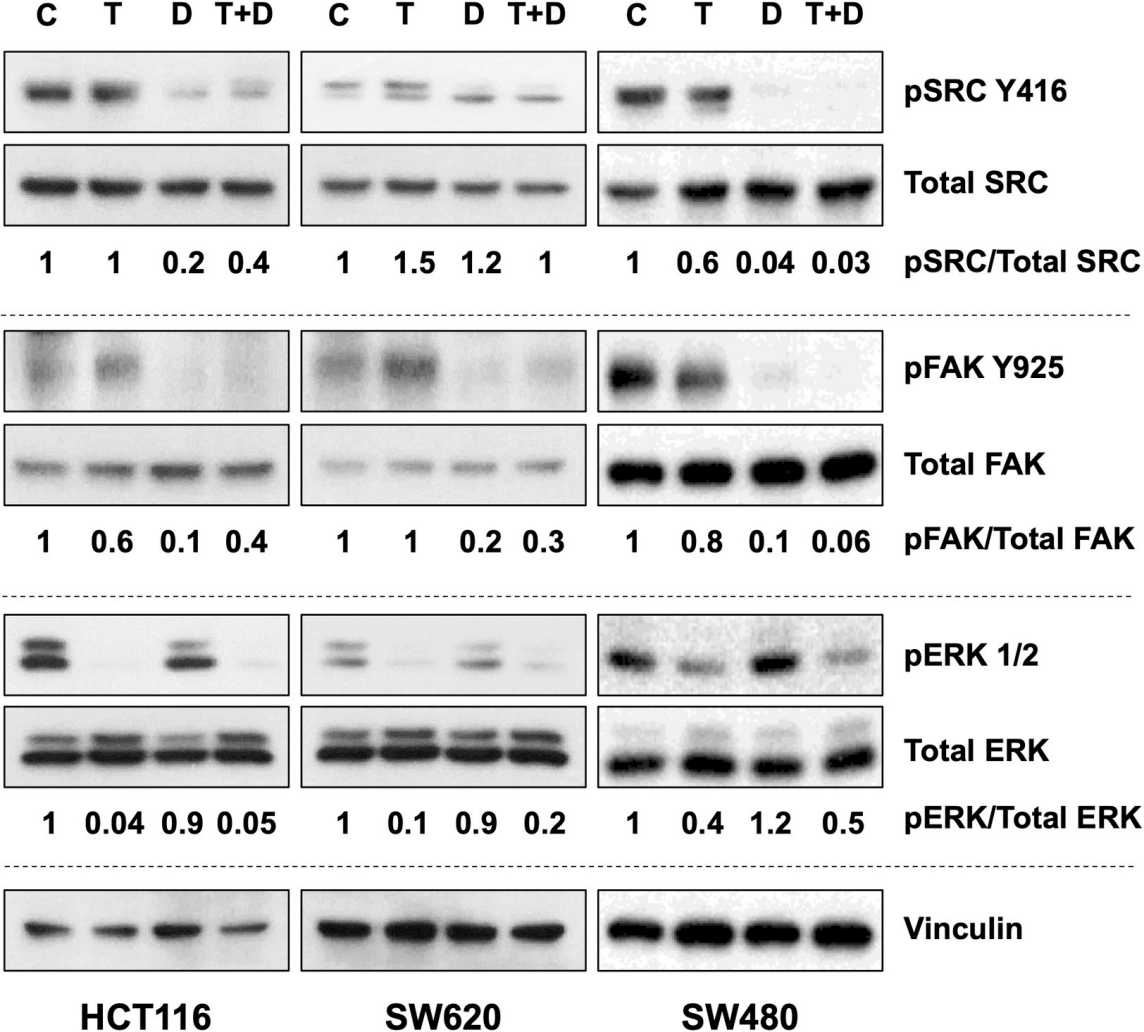
Combined targeting of SRC and MEK inhibits compensatory survival signaling pathways in multiple KRAS-mutated CRC cell lines treated with MEK inhibitors alone. Western blots performed to detect changes in the levels of the signaling factors pSRC, pFAK, and pERK in multiple KRAS-mutated CRC cell lines are shown. Changes in phosphorylation levels were compared with their respective total levels. Vinculin was used as a loading control. C, control; T, trametinib; D, dasatinib; T+D, trametinib and dasatinib. The numbers below the blots denote the phospho-protein levels relative to total protein levels in each sample (Control cells standardized to 1).

We treated HCT116 and SW620 cells with DMSO, trametinib, dasatinib, or the combination of trametinib and dasatinib and performed Western blotting to determine the levels of different signaling factors that are targets of the two drugs. We observed decreased pERK1/2 levels in these cells (Fig 3), indicating target inhibition by trametinib. Notably, we also observed activation of members of the SRC signaling pathway (pSRC and pFAK) in CRC cells treated with trametinib. These increases were abrogated when we treated CRC cells with dasatinib in combination with trametinib.

### Dual inhibition of SRC and MEK enhances levels of apoptotic markers in multiple KRAS-mutated CRC cell lines

**Fig 4:**
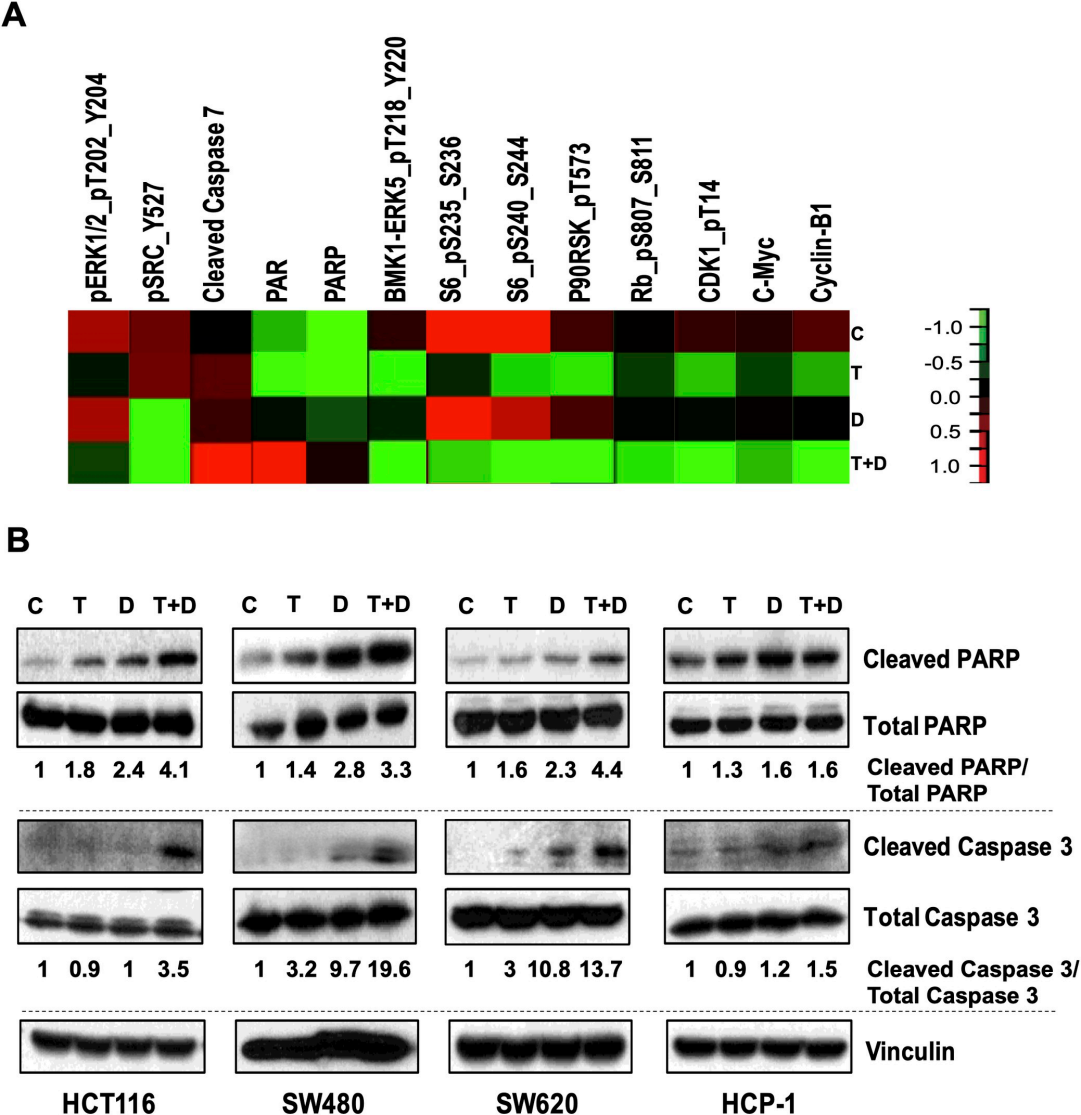
Combined SRC and MEK inhibition alters levels of multiple factors that regulate CRC cell proliferation and enhances apoptotic cell death in multiple KRAS-mutated CRC cell lines. **A,** heat map of results of RPPA analyses done to detect factors that regulate CRC cell proliferation and apoptosis in different KRAS-mutated CRC cell lines following treatment with trametinib (T), dasatinib (D), or both (T+D) for 48 hours. C, control. **B**, Western blots performed to detect the apoptosis markers cleaved PARP, total PARP, cleaved caspase 3, and total caspase 3 in multiple KRAS-mutated CRC cell lines. Vinculin was used as a loading control. The numbers below the blots denote the cleaved protein levels relative to total protein levels in each sample (Control cells standardized to 1).

We determined the effects of combined treatment with dasatinib and trametinib on cell signaling and apoptotic markers in HCT116 cells using an RPPA assay. We observed greater increases in levels of cleaved caspase 7 and PARP in cells treated with the drug combination than in untreated cells or cells treated with dasatinib or trametinib alone (Fig 4A). We also observed greater decreases in levels of the markers of cell proliferation pBMK1-ERK5, pS6, pRb, pCDK1, c-Myc, and cyclin B1. Finally, we observed decreases in levels of pERK and pSRC, which are markers of target inhibition by the two drugs. The RPPA data suggested that this drug combination can reduce the levels of proteins associated with cell proliferation while enhancing markers of apoptosis as compared to dasatinib or trametinib alone.

We also measured induction of apoptosis of CRC cells via Western blotting and flow cytometry. Western blotting showed significantly higher levels of cleaved PARP and cleaved caspase 3 in HCT116, SW480 and SW620 but to a lower extent in HCP-1 cells treated with dasatinib and trametinib than in cells treated with dasatinib or trametinib alone (Fig 4B). Also, in our limited studies, annexin V staining of HCT116, HCP-1, and SW620 cells treated with dasatinib, trametinib, or both showed higher numbers of total apoptotic cells after treatment with the drug combination than after treatment with dasatinib or trametinib alone (S3 Fig).

### Dual inhibition of SRC and MEK does not significantly enhance tumor growth inhibition in multiple KRAS-mutated CRC cell line-derived xenografts or PDXs in vivo

We examined the efficacy of combining dasatinib with trametinib in *in vivo* models of KRAS-mutated CRC. Initially, we performed pilot studies using mice with subcutaneous HCT116 tumors to identify the optimal doses of trametinib and dasatinib that are similar to drug doses used in human patients (S3 Fig). We calculated animal-equivalent doses (AEDs) based on clinical doses of trametinib and dasatinib as described previously [46]. We gave animals the AEDs or two doses higher and lower than the AEDs. Based on our initial results, we determined that 0.2 mg/kg and 10 mg/kg of trametinib and dasatinib, respectively, are the optimum doses for our *in vivo* synergy studies.

**Fig 5:**
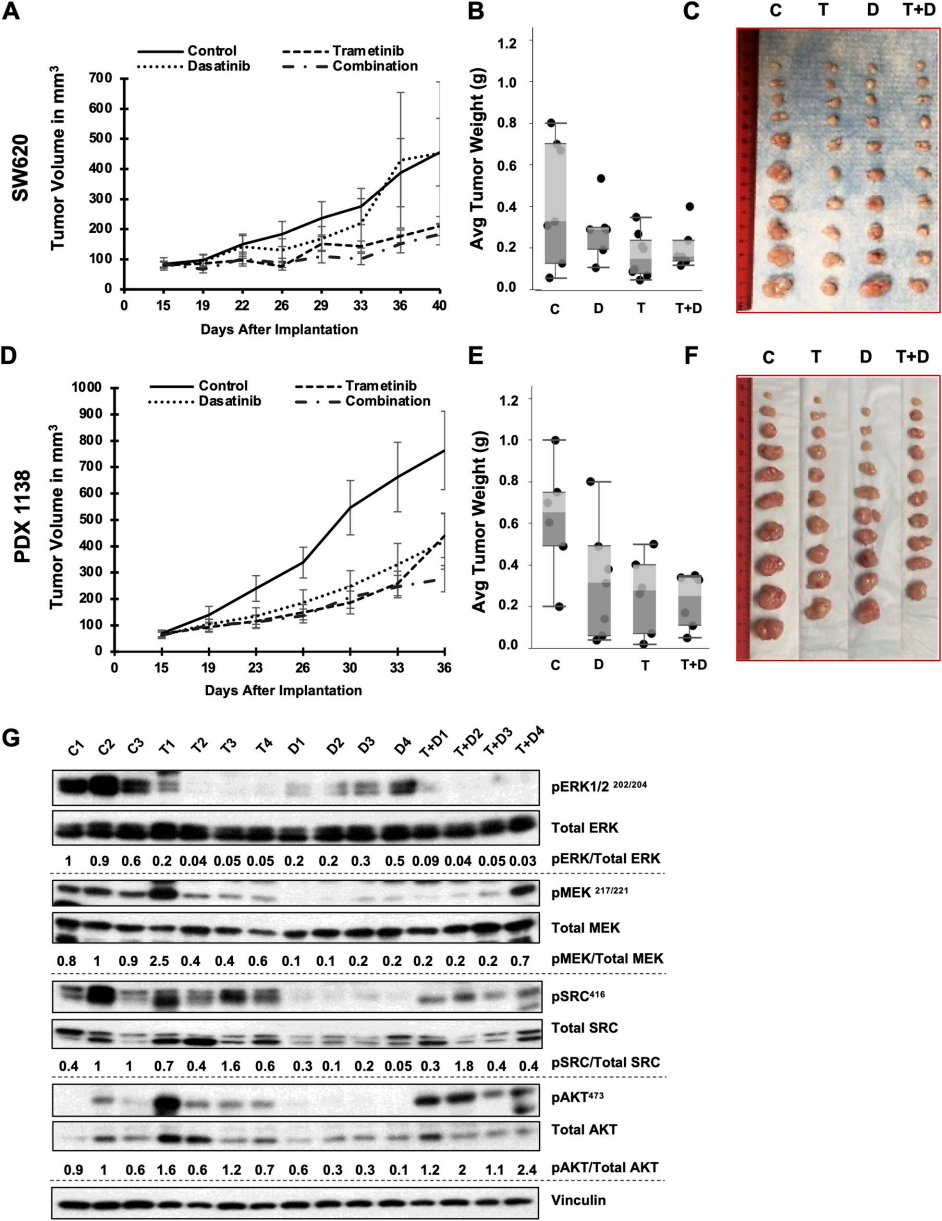
Combination with dasatinib does not significantly enhance the efficacy of trametinib *in vivo*. KRAS-mutated SW620 cells were grown subcutaneously in nude mice and subjected to treatment with vehicle (control), dasatinib (30 mg/kg), trametinib (0.3 mg/kg), or dasatinib and trametinib. KRAS-mutated CRC PDXs (C1138) were implanted subcutaneously in nude mice, which were given vehicle (control), dasatinib (10 mg/kg), trametinib (0.2 mg/kg), or dasatinib and trametinib. Tumor volumes were measured on the days shown on the x-axes (tumors/PDXs implanted on day 0). **A and D,** graphs of tumor growth. **B and E,** average weights of residual tumors plotted at the end of the experiments. **C and F**, images of tumors harvested at end of experiments. Note: no significant differences in either tumor volume or tumor weight between the combination therapy and trametinib groups were observed. **G,** Western blot performed using residual SW620 tumors at the end of *in vivo* experiments to determine the effects of trametinib and dasatinib on target inactivation. Vinculin was used as a loading control for multiple blots measuring different proteins. Only one representative vinculin blot is shown. C1-3, tumors harvested from 3 different control mice; T1-4, tumors harvested from 4 different trametinib treated mice; D1-4, tumors harvested from 4 different dasatinib treated mice; T+D 1–4, tumors harvested from 4 different trametinib and dasatinib treated mice. The numbers below the blots denote the phospho-protein levels relative to total protein levels in each sample (Set of control cells with highest phospho-protein standardized to 1).

We used xenograft tumors generated using the KRAS-mutated CRC cell lines SW620, HCT116, and LS174T for the *in vivo* studies. In these models, treatment with the combination of dasatinib and trametinib produced greater suppression of tumor growth than that in dasatinib-treated and untreated control models (Fig 5A, S5 Fig). However, we found no significant differences in suppression of tumor growth between the models treated with the drug combination and those treated with trametinib alone. Also, we did not observe significant differences in the average xenograft tumor weight at the end of the experiments between the combination treatment group and the dasatinib and trametinib monotherapy groups (Fig 5B, S5 Fig).

We also performed similar *in vivo* studies using KRAS-mutated CRC PDXs. In the C1138 (KRAS G13D) PDX model, the combination of dasatinib and trametinib produced a greater reduction in tumor volume than that in control PDXs but not in PDXs treated with either agent alone (Fig 5D). Similarly, in the C1142 (KRAS G12V) PDX model, the combination of dasatinib and trametinib did not produce significantly greater tumor growth inhibition than did dasatinib or trametinib alone (S5 Fig). Also, the average tumor weight in the combination group did not differ significantly from those in the monotherapy groups at the end of the experiments (Fig 5E, S5 Fig). Representative images of the tumors are shown in Fig 5C, 5F and S6 Fig.

Finally, to determine the molecular effects of dasatinib and trametinib and their combination on target inactivation, we performed Western blotting using protein lysates from residual SW620 xenograft tumors from all of the treatment groups after the end of the treatment regimen. Tumors treated with trametinib, either alone or in combination with dasatinib, had lower pERK1/2 expression than did control or dasatinib-treated tumors (Fig 5G). Dasatinib strongly inhibited levels of pSRC as a single agent. It also produced lower levels of pSRC in combination with trametinib than that in control and trametinib-treated tumors, albeit not to the same extent as in tumors treated with dasatinib only. Of note, the levels of pAKT were markedly higher in the combination group than in the control and single-agent groups.

## Discussion

About 50% of patients with mCRC harbor RAS (KRAS or NRAS) mutations. However, targeting KRAS mutations directly has been ineffective, and efforts at targeting MEK, a downstream mediator of RAS, have led to treatment resistance through multiple resistance mechanisms, especially in CRC patients. Thus, multiple studies have examined the efficacy of combining MEK inhibitors with PI3K/AKT/mTOR [25, 47], Bcl-xL [26], CDK4/6 [27, 28], or autophagy [29–31] inhibitors in preclinical models of various tumors, including CRC. Some of the combinations have made their way to clinical trials. Some of them have not translated into improvements in therapeutic outcomes, and others are in the early stages of clinical evaluation. Thus, to improve the efficacy of MEK inhibitors in patients with RAS-mutated CRC, development of effective combination therapies is urgently needed.

Most combination strategies under investigation rely on preconceived notions of compensatory pathways (e.g., PI3K/AKT inhibitors added to MEK inhibitors [42, 48–51] inhibition of proteins upstream of or downstream from MEK [52]. We hypothesized that MEK-targeted therapy in patients with mCRC will be most effective when combined with other treatments, specifically, either chemotherapy or targeted therapy, that are identified using unbiased screening approaches. In the present study, we performed HTS studies using the MEK inhibitor trametinib (approved by the U.S. Food and Drug Administration for melanoma) as an anchor with two different clinically “ready” compound library sets: 1) the National Cancer Institute oncology set V and 2) a custom clinical set of compounds either approved by the U.S. Food and Drug Administration or in clinical trials. Instead of trying to inhibit targets that could be preconceived (e.g., based on our current understanding of the cellular signaling pathways) and thus biased, our unbiased HTS studies allowed the biology of the CRC cells to determine the best possible combinations that lead to maximum decrease in CRC cell growth. Using the Bliss independence model for synergy in these HTS studies, we identified the SRC inhibitor dasatinib as one of the leading candidates for combination strategies with trametinib based on strong synergistic activity of these two drugs.

Dasatinib, an oral inhibitor of Src family kinases, is a promising therapeutic agent (usually when used in combination with another drug) for the treatment of several cancer types, including chronic myelogenous leukemia, non-small cell lung cancer, small cell lung cancer, advanced breast cancer, pancreatic cancer, prostate cancer, and head and neck squamous cell carcinoma [53]. However, monotherapy with dasatinib in not effective against CRC [54].

Following our initial HTS studies, we validated our findings by examining the combination of dasatinib and trametinib using different *in vitro* approaches with multiple KRAS-mutant CRC cell lines. The ability of a number of these cell lines to form colonies after exposure to the combination was markedly decreased, suggesting that inhibition of these targets leads to a cytotoxic phenotype (Fig 2A). Also, measurement of cell proliferation via MTT assay showed decreased cell survival following the combination treatment (Fig 2B). in addition, we evaluated changes in different proteins involved in multiple signaling networks in CRC cells following treatment with the drug combination. We observed activation of various members of the SRC signaling pathway in CRC cells treated with trametinib, suggesting a possible bypass survival mechanism following MEK inhibition. However, a reduction in the levels of pSRC and pFAK were observed in CRC cells after treatment with dasatinib combined with trametinib (Fig 3). RPPA analyses were performed with HCT116 cells treated with vehicle, trametinib, dasatinib or combination of drugs. RPPA analyses of the combination treatment group indicated to a decrease in various markers of cell proliferation including pERK, pSRC, pBMK1-ERK5, pS6, pRb, pCDK1, c-Myc, cyclin B1 and an increase in markers of apoptosis including cleaved caspase 7 and PARP (Fig 4A), as compared to untreated or single agent treated sets. Thus, the RPPA data suggests that the combination of dasatinib and trametinib can reduce cell proliferation and induce apoptosis. These *in vitro* observations were further validated by an increase in the levels of cleaved PARP and cleaved caspase 3 in CRC cells according to Western blot analysis, indicating increased apoptosis following exposure to the drug combination (Fig 4B). Flow cytometric analysis using annexin V also indicated more apoptotic cells in the combination group than in the vehicle and monotherapy groups (S1 Fig). Taken together, the results of our *in vitro* studies demonstrated that the efficacy of trametinib was significantly enhanced by the addition of dasatinib and led to significant increases in the death of KRAS-mutated CRC cells.

To determine the efficacy of this drug combination *in vivo*, we performed studies using KRAS-mutated CRC cell lines and PDXs. We ensured that the drug doses used in mice were equivalent to those used in humans so that their efficacy closely resembled the possible efficacy achievable in the clinic. We determined the AEDs as described previously [46] and based on previous reports demonstrating evidence of drug doses that effectively led to target inhibition. However, contrary to our *in vitro* data, the combination of dasatinib and trametinib did not have significantly greater effects on tumor growth inhibition or tumor weight reduction than did trametinib alone (Fig 5, S2 Fig). Only tumors generated from LS174T CRC cells showed a slight trend toward tumor size reduction in the presence of the drug combination compared to single agents. These observations were true for treatment strategies using either 0.2 mg/kg trametinib and 10 mg/kg dasatinib based on our pilot studies performed to determine optimal drug dosing or using 0.3 mg/kg trametinib and 30 mg/kg dasatinib, which were based on the AEDs similar to the clinical maximum tolerated doses of these drugs. Notably, end point analysis of proteins from residual tumors using Western blotting showed decreased pERK and pMEK levels in tumors treated with trametinib and decreased pSRC levels in tumors treated with dasatinib, suggesting that the drugs successfully targeted MEK and SRC, respectively. However, the combination treatment failed to reduce tumor growth significantly as compared to the monotherapies. Additionally, residual tumors treated with the drug combination exhibited an increase in the level of pAKT. Note: The drug combination did not increase the levels of pAKT *in vitro* (data not shown). Activation of this compensatory survival pathway likely provides a mechanism of the unexpected failure of the drug combination to enhance tumor growth inhibition *in vivo* (Fig 5E). However, the underlying mechanisms leading to such compensatory pathway activation *in vivo* remain yet unknown. One of the shortcomings of our study is that the roles of other cell types in the tumor microenvironment (TME) including fibroblasts and immune cells on reducing the efficacy of the drug combination have not been elucidated. Previous studies in BRAF mutated melanoma have demonstrated that stromal fibroblast mediated compensatory activation of the AKT pathway leads to reduced efficacy of BRAF inhibitors [55, 56]. It is possible that similar mechanisms lead to reduced efficacy in tumors treated with trametinib and dasatinib. If these cells do play important roles in reducing the efficacy of the trametinib and dasatinib combination, more complex drug combinations may have to be utilized to block the negative roles of these cells in the TME.

Researchers have examined the effects of the combination of SRC and MEK inhibitors on other cancer types with activation of the MAPK pathway [57–59]. Initial success of these studies provides a rationale for combining SRC inhibitors with MEK inhibitors in clinical studies [45, 60, 61]. This rationale is also supported by our *in vitro* studies that identifies dasatinib as a drug that enhances the efficacy of trametinib against KRAS-mutated CRC. Although, our studies demonstrate significant drug synergy *in vitro*, failing to replicate it *in vivo* using multiple KRAS-mutated CRC cell lines and PDXs highlight the importance of careful *in vivo* validation studies in preclinical settings. Also, these studies underscore that preclinical validation studies should be done with drug doses reflecting therapeutic doses used in humans. We believe that it is important to report and publish negative studies where promising *in vitro* studies do not directly translate to success in more advanced in *vivo studies* with clinically relevant dosing of drugs. Publication of negative studies will likely save other investigators time and money, and possibly even provide a stimulus to better understand pharmacology *in vivo* and prevent rapid but futile progression to clinical trials.

In this study we examined the efficacy of combining the MEK inhibitor trametinib with the SRC inhibitor dasatinib to show that although this combination is synergistic *in vitro*, the effects are less significant *in vivo*. While other MEK and SRC inhibitors that are available were not examined, our studies did indicate that the drugs we used inhibited the intended targets. Thus, it is likely that using clinically relevant doses of other MEK and SRC inhibitors will produce similar results. Also, a recent study indicated that CRC tumor with mutations in the G12 residue of KRAS may not benefit from combining SRC and MEK inhibitors [61]. However, our *in vivo* studies with the CRC cell line HCT116 (KRAS G13D) and PDX C1138 (KRAS G13D), both failed to demonstrate improve efficacy of combining MEK and SRC inhibitors. Thus, we conclude that our studies strongly suggest that combining MEK and SRC inhibitors may not provide any additional benefit to patients with KRAS mutated CRC as compared to MEK targeting alone.

In summary, we performed unbiased HTS, identifying that the SRC inhibitor dasatinib is synergistic with the MEK inhibitor trametinib in treating KRAS-mutant CRC. We found the combinatorial effects of these two drugs to be synergistic, leading to significant cell growth inhibition, decreased cell viability, and increased apoptosis *in vitro*. However, our *in vivo* studies using drug doses similar to doses achievable in the clinic failed to demonstrate significantly greater tumor growth-inhibitory effects of the drug combination than those of single-agent trametinib. Hence, our studies demonstrated that the combination of trametinib and dasatinib will be ineffective for most RAS mutated tumors with rare exceptions and the biomarkers identifying mediators of the resistance are unknown.

## Supporting information

S1 Checklist The ARRIVE guidelines 2.0: Author checklist. (PDF)

S1 Fig Statistical results of the 2D HTS assays. **A**, high-throughput assay statistical results. The statistical data demonstrated a high Z’, which was calculated for 16 positive (SN38 [100 nM]) and 16 negative (DMSO) controls. The minimum significant ratio (MSR) was calculated from an eight-point dose response curve tested in duplicate on each assay plate. **B**, graph of the individual Z’ values for each plate. The data demonstrated high consistency in the assay and no outliers. **C**, representative dose response curves for each assay plate. The data demonstrated high reproducibility of dose response data and no assay drift.
(TIF)

S2 Fig Bliss calculations for colony formation assays. Clonogenic analysis demonstrated synergy of SRC and MEK inhibitors in multiple KRAS-mutated CRC cell lines. Colony formation assays were performed with multiple KRAS-mutated CRC cell lines. Cells were treated for 7 days and stained with a 0.5% methylene blue solution. Methylene blue incorporated in CRC cells was extracted in 1% SDS, and absorbance of the extracted dye was measured using a plate reader at 570 nM. All data are presented as mean (± SD) values. A Bliss additivity model was used to calculate excess over Bliss.
(TIF)

S3 Fig Combining trametinib with dasatinib increases apoptosis in CRC cells *in vitro*. Flow cytometry was performed to determine the effects of combined SRC and MEK inhibition on enhancement of apoptotic CRC cell death. HCT116, HCP-1, and SW620 cells were treated with trametinib, dasatinib, or both for 72 hours. Cells were washed and stained for annexin V and propidium iodide for 15 minutes at room temperature. The percentages of apoptotic cells are shown in their respective quadrants. Q2, apoptotic cells; Q4, early apoptotic cells.
(TIF)

S4 Fig Determination of the *in vivo* treatment doses of trametinib and dasatinib. KRAS-mutated HCT116 cells were grown subcutaneously in mice. **A,** average tumor volumes in animals given a vehicle (control) or trametinib (0.1, 0.3, or 1.0 mg/kg; 5 days/week). **B,** average tumor volumes in animals given a vehicle (control) or dasatinib (10, 25, or 50 mg/kg; 5 days/week). Tumor volumes were measured on the days shown on the x-axes (tumors cells implanted on day 0). Note: in this pilot study, each treatment arm had three or four animals. Thus, statistical calculations are not shown.
(TIF)

S5 Fig Efficacy of combining an SRC inhibitor with an MEK inhibitor *in vivo*. KRAS-mutated CRC cells (HCT116 and LS174T) and CRC PDXs (C1142) were grown subcutaneously in mice, which were given a vehicle (control), dasatinib (10 mg/kg), trametinib (0.2 mg/kg), or dasatinib and trametinib. Tumor volumes were measured on the days shown on the x-axes (tumors implanted on day 0). **A, C, and E,** graphs of tumor growth. **B, D, and F,** average weights of residual tumors at the end of the experiments. Note: no significant differences in tumor volume or tumor weight between the combination therapy and trametinib groups were observed.
(TIF)

S6 Fig Effect of single-agent and combination treatment with trametinib and dasatinib on tumor growth. KRAS-mutated CRC cells (LS174T, and HCT116) and CRC PDX (C1142) were grown subcutaneously in mice, which were given a vehicle (C), trametinib (T), dasatinib (D), or trametinib and dasatinib (T+D). Photographs of tumors harvested at the end of the experiments are shown. The cell line and PDX names are shown at the bottom of each panel. Note: all experiments were initiated with 10 mice per group. Some experimental animals died or were euthanized for causes unrelated to treatment during the course of the studies.
(TIF)

S1 Raw images (PDF)

## References

[1] American Cancer Society. Cancer facts & figures. Atlanta, GA: The Society. p. v.

[2] Douillard JY, Oliner KS, Siena S, Tabernero J, Burkes R, Barugel M, et al. Panitumumab-FOLFOX4 treatment and RAS mutations in colorectal cancer. N Engl J Med. 2013;369(11):1023–34. doi: 10.1056/NEJMoa1305275 24024839

[3] Davies JM, Goldberg RM. Treatment of metastatic colorectal cancer. Semin Oncol. 2011;38(4):552–60. doi: 10.1053/j.seminoncol.2011.05.009 21810514

[4] Le DT, Uram JN, Wang H, Bartlett BR, Kemberling H, Eyring AD, et al. PD-1 Blockade in Tumors with Mismatch-Repair Deficiency. N Engl J Med. 2015;372(26):2509–20. doi: 10.1056/NEJMoa1500596 26028255 PMC4481136

[5] Overman MJ, Lonardi S, Wong KYM, Lenz HJ, Gelsomino F, Aglietta M, et al. Durable Clinical Benefit With Nivolumab Plus Ipilimumab in DNA Mismatch Repair-Deficient/Microsatellite Instability-High Metastatic Colorectal Cancer. J Clin Oncol. 2018:JCO2017769901. doi: 10.1200/JCO.2017.76.9901 29355075

[6] Overman MJ, McDermott R, Leach JL, Lonardi S, Lenz HJ, Morse MA, et al. Nivolumab in patients with metastatic DNA mismatch repair-deficient or microsatellite instability-high colorectal cancer (CheckMate 142): an open-label, multicentre, phase 2 study. Lancet Oncol. 2017;18(9):1182–91. doi: 10.1016/S1470-2045(17)30422-9 28734759 PMC6207072

[7] Eng C, Kim TW, Bendell J, Argiles G, Tebbutt NC, Di Bartolomeo M, et al. Atezolizumab with or without cobimetinib versus regorafenib in previously treated metastatic colorectal cancer (IMblaze370): a multicentre, open-label, phase 3, randomised, controlled trial. Lancet Oncol. 2019;20(6):849–61. doi: 10.1016/S1470-2045(19)30027-0 31003911

[8] Mehrvarz Sarshekeh A, Overman MJ, Kopetz S. Nivolumab in the treatment of microsatellite instability high metastatic colorectal cancer. Future Oncol. 2018;14(18):1869–74. doi: 10.2217/fon-2017-0696 29473436 PMC6088272

[9] Dienstmann R, Vermeulen L, Guinney J, Kopetz S, Tejpar S, Tabernero J. Consensus molecular subtypes and the evolution of precision medicine in colorectal cancer. Nat Rev Cancer. 2017;17(2):79–92. doi: 10.1038/nrc.2016.126 28050011

[10] Guinney J, Dienstmann R, Wang X, de Reynies A, Schlicker A, Soneson C, et al. The consensus molecular subtypes of colorectal cancer. Nat Med. 2015;21(11):1350–6. doi: 10.1038/nm.3967 26457759 PMC4636487

[11] Loree JM, Pereira AAL, Lam M, Willauer AN, Raghav K, Dasari A, et al. Classifying Colorectal Cancer by Tumor Location Rather than Sidedness Highlights a Continuum in Mutation Profiles and Consensus Molecular Subtypes. Clin Cancer Res. 2018;24(5):1062–72. doi: 10.1158/1078-0432.CCR-17-2484 29180604 PMC5844818

[12] Makinen MJ. Colorectal serrated adenocarcinoma. Histopathology. 2007;50(1):131–50. doi: 10.1111/j.1365-2559.2006.02548.x 17204027

[13] Passot G, Kim BJ, Glehen O, Mehran RJ, Kopetz SE, Goere D, et al. Impact of RAS Mutations in Metastatic Colorectal Cancer After Potentially Curative Resection: Does Site of Metastases Matter? Ann Surg Oncol. 2018;25(1):179–87. doi: 10.1245/s10434-017-6141-7 29071660

[14] Loupakis F, Ruzzo A, Cremolini C, Vincenzi B, Salvatore L, Santini D, et al. KRAS codon 61, 146 and BRAF mutations predict resistance to cetuximab plus irinotecan in KRAS codon 12 and 13 wild-type metastatic colorectal cancer. Br J Cancer. 2009;101(4):715–21. doi: 10.1038/sj.bjc.6605177 19603018 PMC2736831

[15] Seshagiri S, Stawiski EW, Durinck S, Modrusan Z, Storm EE, Conboy CB, et al. Recurrent R-spondin fusions in colon cancer. Nature. 2012;488(7413):660–4. doi: 10.1038/nature11282 22895193 PMC3690621

[16] Tol J, Koopman M, Cats A, Rodenburg CJ, Creemers GJ, Schrama JG, et al. Chemotherapy, bevacizumab, and cetuximab in metastatic colorectal cancer. N Engl J Med. 2009;360(6):563–72. doi: 10.1056/NEJMoa0808268 19196673

[17] Van Cutsem E, Kohne CH, Lang I, Folprecht G, Nowacki MP, Cascinu S, et al. Cetuximab plus irinotecan, fluorouracil, and leucovorin as first-line treatment for metastatic colorectal cancer: updated analysis of overall survival according to tumor KRAS and BRAF mutation status. J Clin Oncol. 2011;29(15):2011–9. doi: 10.1200/JCO.2010.33.5091 21502544

[18] Yokota T, Ura T, Shibata N, Takahari D, Shitara K, Nomura M, et al. BRAF mutation is a powerful prognostic factor in advanced and recurrent colorectal cancer. Br J Cancer. 2011;104(5):856–62. doi: 10.1038/bjc.2011.19 21285991 PMC3048210

[19] Rao S, Cunningham D, de Gramont A, Scheithauer W, Smakal M, Humblet Y, et al. Phase III double-blind placebo-controlled study of farnesyl transferase inhibitor R115777 in patients with refractory advanced colorectal cancer. J Clin Oncol. 2004;22(19):3950–7. doi: 10.1200/JCO.2004.10.037 15459217

[20] Ostrem JM, Peters U, Sos ML, Wells JA, Shokat KM. K-Ras(G12C) inhibitors allosterically control GTP affinity and effector interactions. Nature. 2013;503(7477):548–51. doi: 10.1038/nature12796 24256730 PMC4274051

[21] Hallin J, Engstrom LD, Hargis L, Calinisan A, Aranda R, Briere DM, et al. The KRAS(G12C) Inhibitor MRTX849 Provides Insight toward Therapeutic Susceptibility of KRAS-Mutant Cancers in Mouse Models and Patients. Cancer Discov. 2020;10(1):54–71. doi: 10.1158/2159-8290.CD-19-1167 31658955 PMC6954325

[22] Canon J, Rex K, Saiki AY, Mohr C, Cooke K, Bagal D, et al. The clinical KRAS(G12C) inhibitor AMG 510 drives anti-tumour immunity. Nature. 2019;575(7781):217–23. doi: 10.1038/s41586-019-1694-1 31666701

[23] Weiss RDY J., Johnson M.L., Spira A., Klempner S.J., Barve M.A., Christensen J.G., et al. LBA6 KRYSTAL-1: Adagrasib (MRTX849) as monotherapy or combined with cetuximab (Cetux) in patients (Pts) with colorectal cancer (CRC) harboring a KRASG12C mutation. Ann Oncol. 2021;32:S1294.

[24] Infante JR, Fecher LA, Falchook GS, Nallapareddy S, Gordon MS, Becerra C, et al. Safety, pharmacokinetic, pharmacodynamic, and efficacy data for the oral MEK inhibitor trametinib: a phase 1 dose-escalation trial. Lancet Oncol. 2012;13(8):773–81. doi: 10.1016/S1470-2045(12)70270-X 22805291

[25] Do K, Speranza G, Bishop R, Khin S, Rubinstein L, Kinders RJ, et al. Biomarker-driven phase 2 study of MK-2206 and selumetinib (AZD6244, ARRY-142886) in patients with colorectal cancer. Invest New Drugs. 2015;33(3):720–8. doi: 10.1007/s10637-015-0212-z 25637165 PMC7709950

[26] Corcoran RB, Cheng KA, Hata AN, Faber AC, Ebi H, Coffee EM, et al. Synthetic lethal interaction of combined BCL-XL and MEK inhibition promotes tumor regressions in KRAS mutant cancer models. Cancer Cell. 2013;23(1):121–8. doi: 10.1016/j.ccr.2012.11.007 23245996 PMC3667614

[27] Lee MS, Helms TL, Feng N, Gay J, Chang QE, Tian F, et al. Efficacy of the combination of MEK and CDK4/6 inhibitors in vitro and in vivo in KRAS mutant colorectal cancer models. Oncotarget. 2016;7(26):39595–608. doi: 10.18632/oncotarget.9153 27167191 PMC5129956

[28] Pek M, Yatim S, Chen Y, Li J, Gong M, Jiang X, et al. Oncogenic KRAS-associated gene signature defines co-targeting of CDK4/6 and MEK as a viable therapeutic strategy in colorectal cancer. Oncogene. 2017;36(35):4975–86. doi: 10.1038/onc.2017.120 28459468

[29] Lee CS, Lee LC, Yuan TL, Chakka S, Fellmann C, Lowe SW, et al. MAP kinase and autophagy pathways cooperate to maintain RAS mutant cancer cell survival. Proc Natl Acad Sci U S A. 2019;116(10):4508–17. doi: 10.1073/pnas.1817494116 30709910 PMC6410784

[30] Bryant KL, Stalnecker CA, Zeitouni D, Klomp JE, Peng S, Tikunov AP, et al. Combination of ERK and autophagy inhibition as a treatment approach for pancreatic cancer. Nat Med. 2019;25(4):628–40. doi: 10.1038/s41591-019-0368-8 30833752 PMC6484853

[31] Kinsey CG, Camolotto SA, Boespflug AM, Guillen KP, Foth M, Truong A, et al. Publisher Correction: Protective autophagy elicited by RAF—>MEK—>ERK inhibition suggests a treatment strategy for RAS-driven cancers. Nat Med. 2019;25(5):861. 10.1038/s41591-019-0433-3 30918364

[32] Fan F, Bellister S, Lu J, Ye X, Boulbes DR, Tozzi F, et al. The requirement for freshly isolated human colorectal cancer (CRC) cells in isolating CRC stem cells. Br J Cancer. 2015;112(3):539–46. doi: 10.1038/bjc.2014.620 25535733 PMC4453647

[33] Wang R, Bhattacharya R, Ye X, Fan F, Boulbes DR, Ellis LM. Endothelial Cells Promote Colorectal Cancer Cell Survival by Activating the HER3-AKT Pathway in a Paracrine Fashion. Mol Cancer Res. 2019;17(1):20–9. doi: 10.1158/1541-7786.MCR-18-0341 30131447 PMC6318043

[34] Bhattacharya R, Cabral F. Molecular basis for class V beta-tubulin effects on microtubule assembly and paclitaxel resistance. J Biol Chem. 2009;284(19):13023–32. doi: 10.1074/jbc.M900167200 19282281 PMC2676035

[35] Bhattacharya R, Fan F, Wang R, Ye X, Xia L, Boulbes D, et al. Intracrine VEGF signalling mediates colorectal cancer cell migration and invasion. Br J Cancer. 2017;117(6):848–55. doi: 10.1038/bjc.2017.238 28742793 PMC5589988

[36] Bhattacharya R, Ye XC, Wang R, Ling X, McManus M, Fan F, et al. Intracrine VEGF Signaling Mediates the Activity of Prosurvival Pathways in Human Colorectal Cancer Cells. Cancer Res. 2016;76(10):3014–24. doi: 10.1158/0008-5472.CAN-15-1605 26988990 PMC4873444

[37] Ghosh S, Prasad M, Kundu K, Cohen L, Yegodayev KM, Zorea J, et al. Tumor Tissue Explant Culture of Patient-Derived Xenograft as Potential Prioritization Tool for Targeted Therapy. Front Oncol. 2019;9:17. doi: 10.3389/fonc.2019.00017 30723707 PMC6350270

[38] Lu J, Ye X, Fan F, Xia L, Bhattacharya R, Bellister S, et al. Endothelial cells promote the colorectal cancer stem cell phenotype through a soluble form of Jagged-1. Cancer Cell. 2013;23(2):171–85. doi: 10.1016/j.ccr.2012.12.021 23375636 PMC3574187

[39] Handley KF, Rodriguez-Aguayo C, Ma S, Stur E, Joseph R, Bayraktar E, et al. Rational Combination of CRM1 Inhibitor Selinexor and Olaparib Shows Synergy in Ovarian Cancer Cell Lines and Mouse Models. Mol Cancer Ther. 2021;20(12):2352–61. doi: 10.1158/1535-7163.MCT-21-0370 34583979 PMC8643313

[40] Zhao W, Sachsenmeier K, Zhang L, Sult E, Hollingsworth RE, Yang H. A New Bliss Independence Model to Analyze Drug Combination Data. Journal of Biomolecular Screening. 2014;19(5):817–21. doi: 10.1177/1087057114521867 24492921

[41] Henderson YC, Mohamed ASR, Maniakas A, Chen Y, Powell RT, Peng S, et al. A High-throughput Approach to Identify Effective Systemic Agents for the Treatment of Anaplastic Thyroid Carcinoma. J Clin Endocrinol Metab. 2021;106(10):2962–78. doi: 10.1210/clinem/dgab424 34120183 PMC8475220

[42] Grilley-Olson JE, Bedard PL, Fasolo A, Cornfeld M, Cartee L, Razak AR, et al. A phase Ib dose-escalation study of the MEK inhibitor trametinib in combination with the PI3K/mTOR inhibitor GSK2126458 in patients with advanced solid tumors. Invest New Drugs. 2016;34(6):740–9. doi: 10.1007/s10637-016-0377-0 27450049 PMC7574157

[43] Kim SY, Jeong EH, Lee TG, Kim HR, Kim CH. The Combination of Trametinib, a MEK Inhibitor, and Temsirolimus, an mTOR Inhibitor, Radiosensitizes Lung Cancer Cells. Anticancer Res. 2021;41(6):2885–94. doi: 10.21873/anticanres.15070 34083279

[44] Luo J, Makhnin A, Tobi Y, Ahn L, Hayes SA, Iqbal A, et al. Erlotinib and Trametinib in Patients With EGFR-Mutant Lung Adenocarcinoma and Acquired Resistance to a Prior Tyrosine Kinase Inhibitor. JCO Precis Oncol. 2021;5. doi: 10.1200/PO.20.00315 34250388 PMC8232136

[45] Dawson JC, Munro A, Macleod K, Muir M, Timpson P, Williams RJ, et al. Pathway profiling of a novel SRC inhibitor, AZD0424, in combination with MEK inhibitors for cancer treatment. Mol Oncol. 2022;16(5):1072–90. doi: 10.1002/1878-0261.13151 34856074 PMC8895456

[46] Nair AB, Jacob S. A simple practice guide for dose conversion between animals and human. J Basic Clin Pharm. 2016;7(2):27–31. 27057123 10.4103/0976-0105.177703 PMC4804402

[47] Pitts TM, Newton TP, Bradshaw-Pierce EL, Addison R, Arcaroli JJ, Klauck PJ, et al. Dual pharmacological targeting of the MAP kinase and PI3K/mTOR pathway in preclinical models of colorectal cancer. PLoS One. 2014;9(11):e113037. doi: 10.1371/journal.pone.0113037 25401499 PMC4234626

[48] Wong CH, Ma BB, Cheong HT, Hui CW, Hui EP, Chan AT. Preclinical evaluation of PI3K inhibitor BYL719 as a single agent and its synergism in combination with cisplatin or MEK inhibitor in nasopharyngeal carcinoma (NPC). Am J Cancer Res. 2015;5(4):1496–506. 26101713 PMC4473326

[49] Shapiro GI, LoRusso P, Kwak E, Pandya S, Rudin CM, Kurkjian C, et al. Phase Ib study of the MEK inhibitor cobimetinib (GDC-0973) in combination with the PI3K inhibitor pictilisib (GDC-0941) in patients with advanced solid tumors. Invest New Drugs. 2020;38(2):419–32. doi: 10.1007/s10637-019-00776-6 31020608

[50] Ramanathan RK, Von Hoff DD, Eskens F, Blumenschein G, Jr., Richards D, Genvresse I, et al. Phase Ib Trial of the PI3K Inhibitor Copanlisib Combined with the Allosteric MEK Inhibitor Refametinib in Patients with Advanced Cancer. Target Oncol. 2020;15(2):163–74. doi: 10.1007/s11523-020-00714-0 32314268 PMC7591420

[51] Goulielmaki M, Assimomytis N, Rozanc J, Taki E, Christodoulou I, Alexopoulos LG, et al. DPS-2: A Novel Dual MEK/ERK and PI3K/AKT Pathway Inhibitor with Powerful Ex Vivo and In Vivo Anticancer Properties. Transl Oncol. 2019;12(7):932–50. doi: 10.1016/j.tranon.2019.04.005 31096110 PMC6520640

[52] Zhang YJ, Tian XQ, Sun DF, Zhao SL, Xiong H, Fang JY. Combined inhibition of MEK and mTOR signaling inhibits initiation and progression of colorectal cancer. Cancer Invest. 2009;27(3):273–85. doi: 10.1080/07357900802314893 19194827

[53] Kim DW, Goh YT, Hsiao HH, Caguioa PB, Kim D, Kim WS, et al. Clinical profile of dasatinib in Asian and non-Asian patients with chronic myeloid leukemia. Int J Hematol. 2009;89(5):664–72. doi: 10.1007/s12185-009-0326-1 19455391

[54] Sharma MR, Wroblewski K, Polite BN, Knost JA, Wallace JA, Modi S, et al. Dasatinib in previously treated metastatic colorectal cancer: a phase II trial of the University of Chicago Phase II Consortium. Invest New Drugs. 2012;30(3):1211–5. doi: 10.1007/s10637-011-9681-x 21552992 PMC4317401

[55] Straussman R, Morikawa T, Shee K, Barzily-Rokni M, Qian ZR, Du J, et al. Tumour micro-environment elicits innate resistance to RAF inhibitors through HGF secretion. Nature. 2012;487(7408):500–4. doi: 10.1038/nature11183 22763439 PMC3711467

[56] Fedorenko IV, Wargo JA, Flaherty KT, Messina JL, Smalley KSM. BRAF Inhibition Generates a Host-Tumor Niche that Mediates Therapeutic Escape. J Invest Dermatol. 2015;135(12):3115–24. doi: 10.1038/jid.2015.329 26302068 PMC4648653

[57] Yuan M, Xu LF, Zhang J, Kong SY, Wu M, Lao YZ, et al. SRC and MEK Co-inhibition Synergistically Enhances the Anti-tumor Effect in Both Non-small-cell Lung Cancer (NSCLC) and Erlotinib-Resistant NSCLC. Front Oncol. 2019;9:586. doi: 10.3389/fonc.2019.00586 31428570 PMC6689998

[58] Simpkins F, Jang K, Yoon H, Hew KE, Kim M, Azzam DJ, et al. Dual Src and MEK Inhibition Decreases Ovarian Cancer Growth and Targets Tumor Initiating Stem-Like Cells. Clin Cancer Res. 2018;24(19):4874–86. doi: 10.1158/1078-0432.CCR-17-3697 29959144 PMC6557165

[59] Ferguson J, Arozarena I, Ehrhardt M, Wellbrock C. Combination of MEK and SRC inhibition suppresses melanoma cell growth and invasion. Oncogene. 2013;32(1):86–96. doi: 10.1038/onc.2012.25 22310287 PMC3378628

[60] Davis TB, Gupta S, Yang M, Pflieger L, Rajan M, Wang H, et al. Ras Pathway Activation and MEKi Resistance Scores Predict the Efficiency of MEKi and SRCi Combination to Induce Apoptosis in Colorectal Cancer. Cancers (Basel). 2022;14(6). doi: 10.3390/cancers14061451 35326598 PMC8945886

[61] Gavert N, Zwang Y, Weiser R, Greenberg O, Halperin S, Jacobi O, et al. Ex vivo organotypic cultures for synergistic therapy prioritization identify patient-specific responses to combined MEK and Src inhibition in colorectal cancer. Nat Cancer. 2022;3(2):219–31. doi: 10.1038/s43018-021-00325-2 35145327

