# Supplementary figures and images for: Combining MEK and SRC inhibitors for treatment of colorectal cancer demonstrate increased efficacy in vitro but not in vivo
Source: PLoS One. 2023 Mar 23;18(3):e0281063. doi: 10.1371/journal.pone.0281063 (PMC10035898; doi:10.1371/journal.pone.0281063)

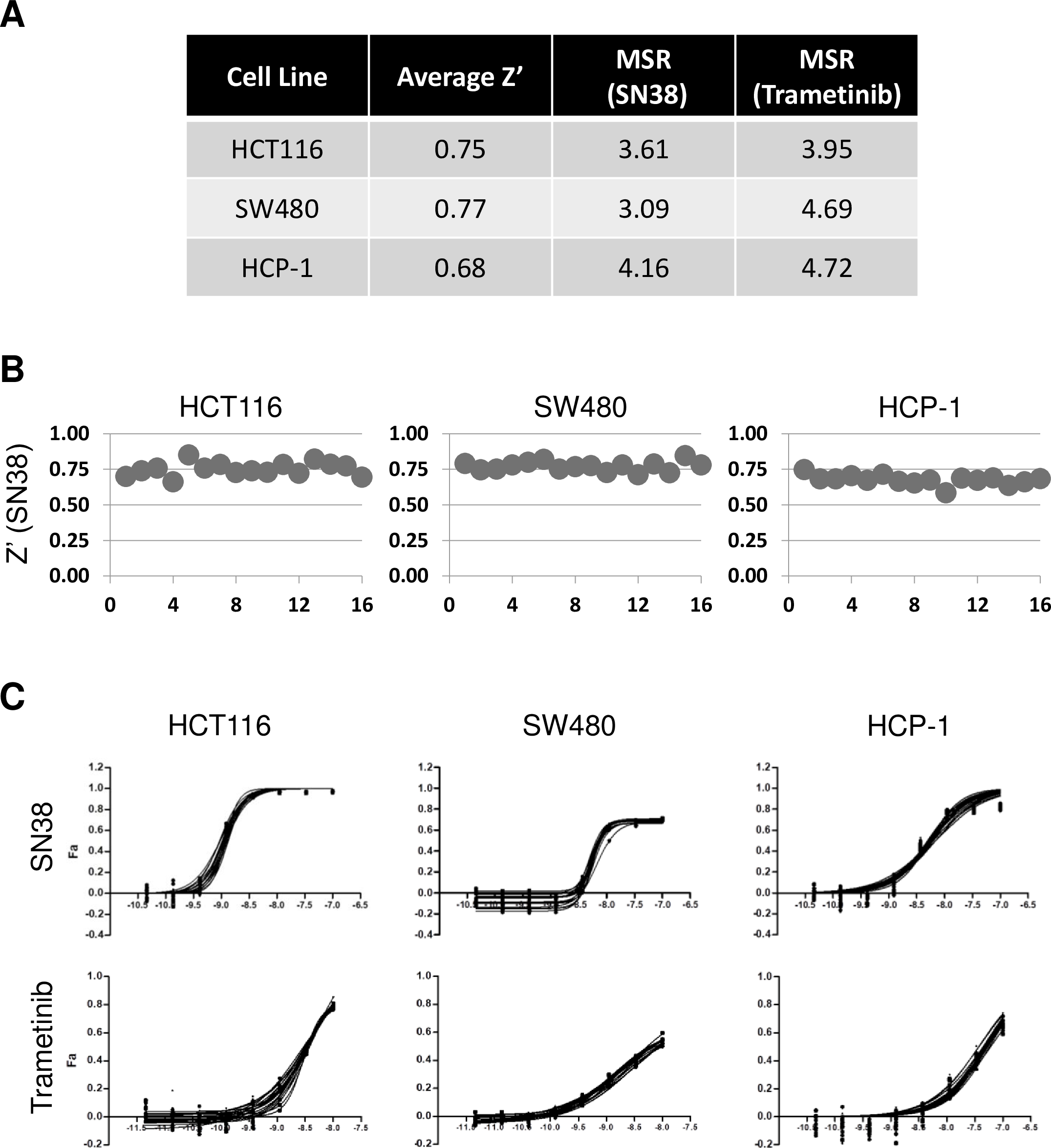

Supplement: S1 Fig — A, high-throughput assay statistical results. The statistical data demonstrated a high Z’, which was calculated for 16 positive (SN38 [100 nM]) and 16 negative (DMSO) controls. The minimum significant ratio (MSR) was calculated from an eight-point dose response curve tested in duplicate on each assay plate. B, graph of the individual Z’ values for each plate. The data demonstrated high consistency in the assay and no outliers. C, representative dose response curves for each assay plate. The data demonstrated high reproducibility of dose response data and no assay drift. (TIF) [file pone.0281063.s002.tif]

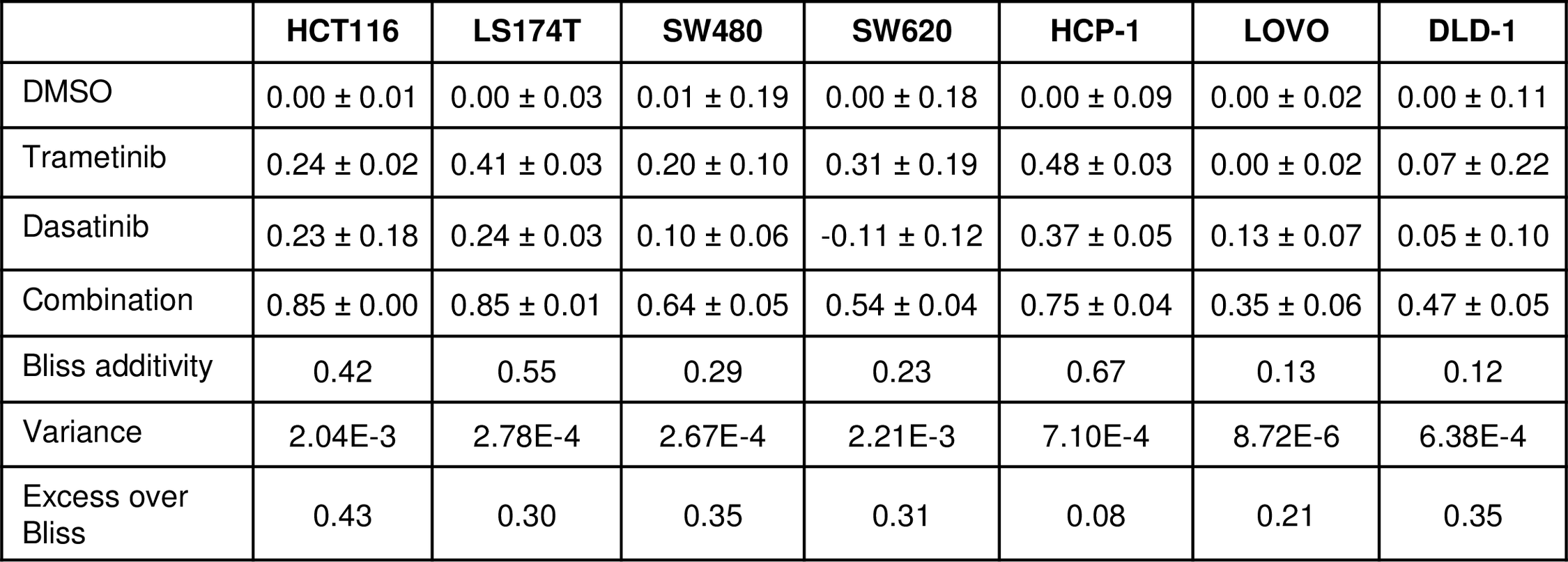

Supplement: S2 Fig — Clonogenic analysis demonstrated synergy of SRC and MEK inhibitors in multiple KRAS-mutated CRC cell lines. Colony formation assays were performed with multiple KRAS-mutated CRC cell lines. Cells were treated for 7 days and stained with a 0.5% methylene blue solution. Methylene blue incorporated in CRC cells was extracted in 1% SDS, and absorbance of the extracted dye was measured using a plate reader at 570 nM. All data are presented as mean (± SD) values. A Bliss additivity model was used to calculate excess over Bliss. (TIF) [file pone.0281063.s003.tif]

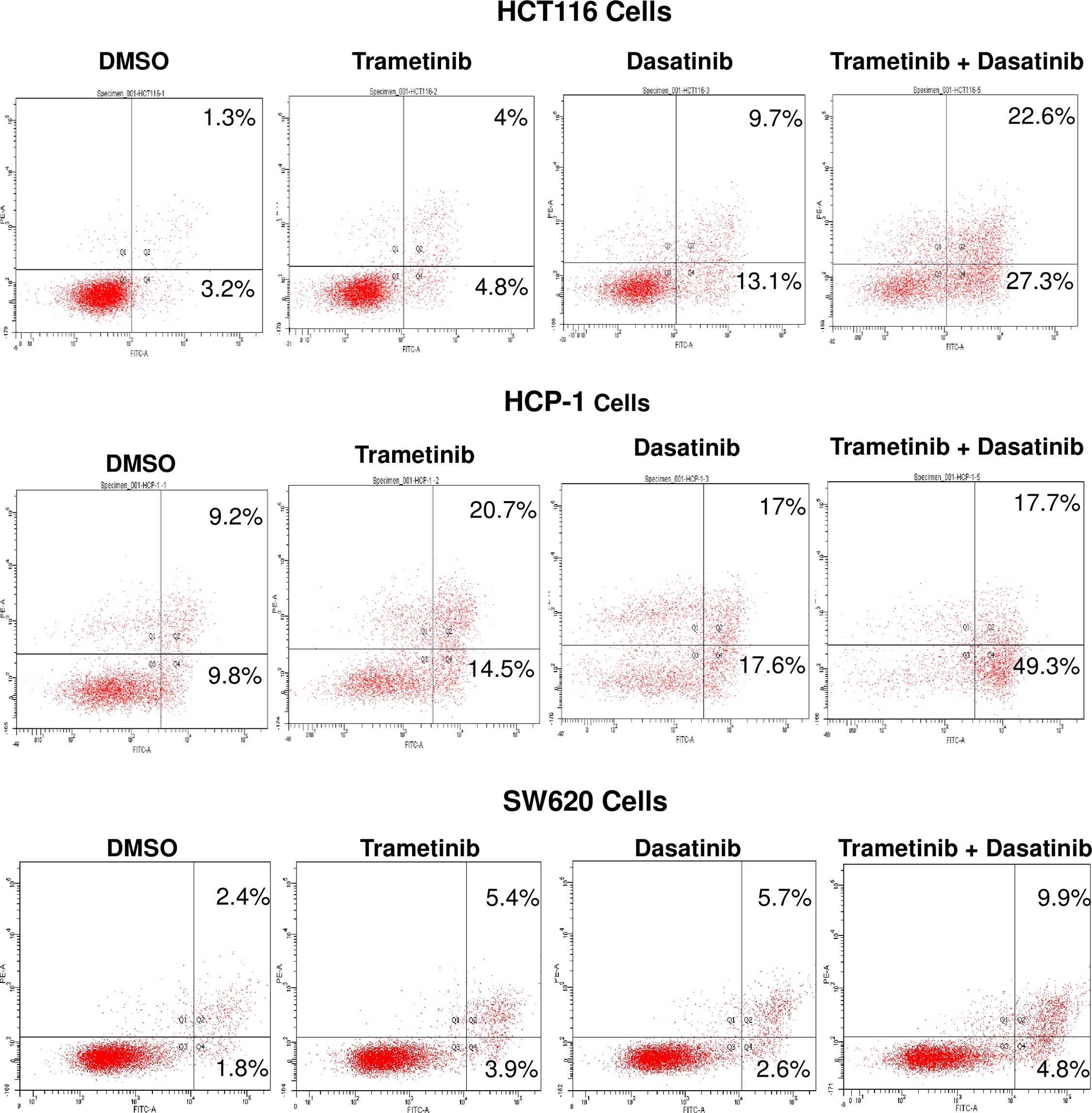

Supplement: S3 Fig — Flow cytometry was performed to determine the effects of combined SRC and MEK inhibition on enhancement of apoptotic CRC cell death. HCT116, HCP-1, and SW620 cells were treated with trametinib, dasatinib, or both for 72 hours. Cells were washed and stained for annexin V and propidium iodide for 15 minutes at room temperature. The percentages of apoptotic cells are shown in their respective quadrants. Q2, apoptotic cells; Q4, early apoptotic cells. (TIF) [file pone.0281063.s004.tif]

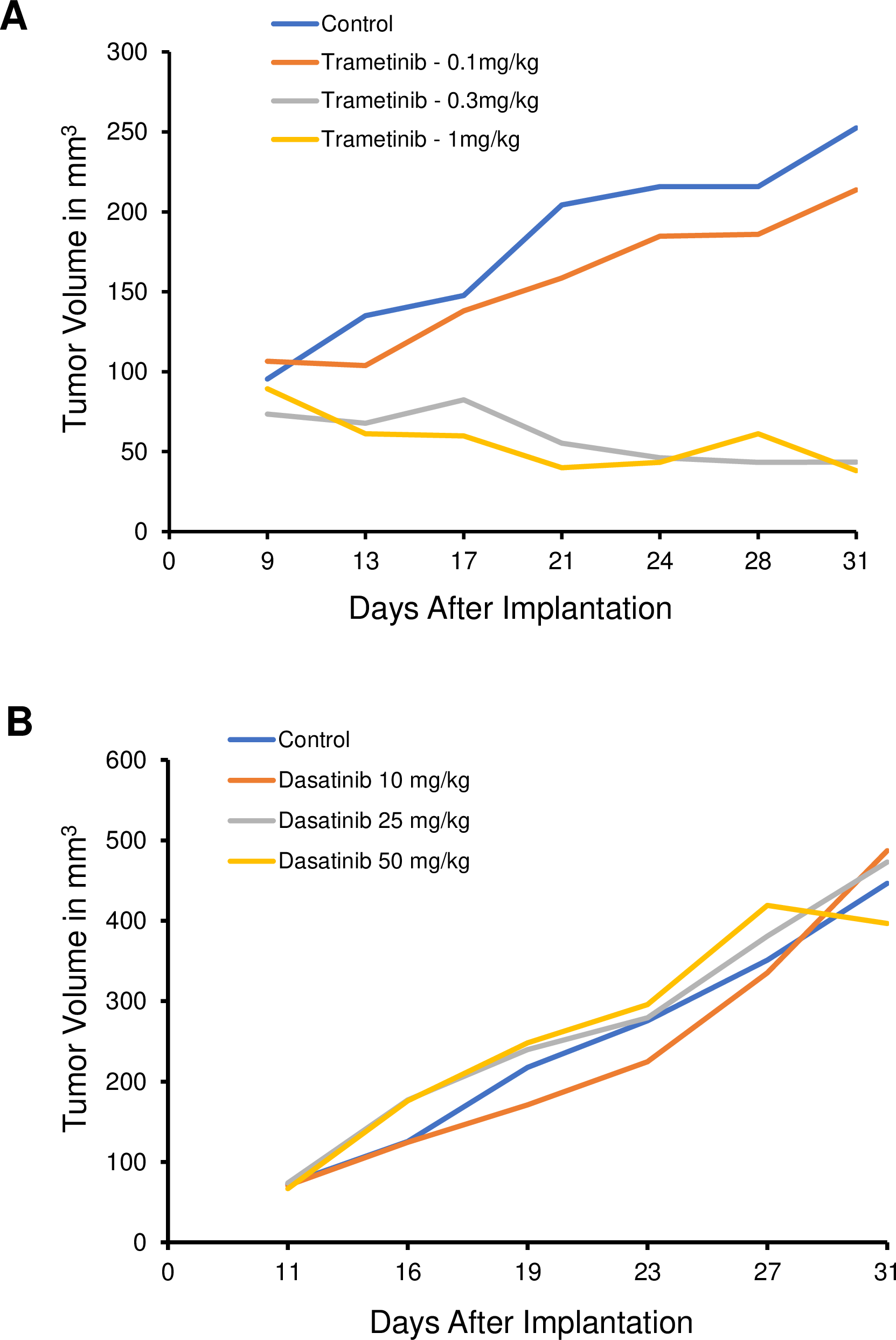

Supplement: S4 Fig — KRAS-mutated HCT116 cells were grown subcutaneously in mice. A, average tumor volumes in animals given a vehicle (control) or trametinib (0.1, 0.3, or 1.0 mg/kg; 5 days/week). B, average tumor volumes in animals given a vehicle (control) or dasatinib (10, 25, or 50 mg/kg; 5 days/week). Tumor volumes were measured on the days shown on the x-axes (tumors cells implanted on day 0). Note: in this pilot study, each treatment arm had three or four animals. Thus, statistical calculations are not shown. (TIF) [file pone.0281063.s005.tif]

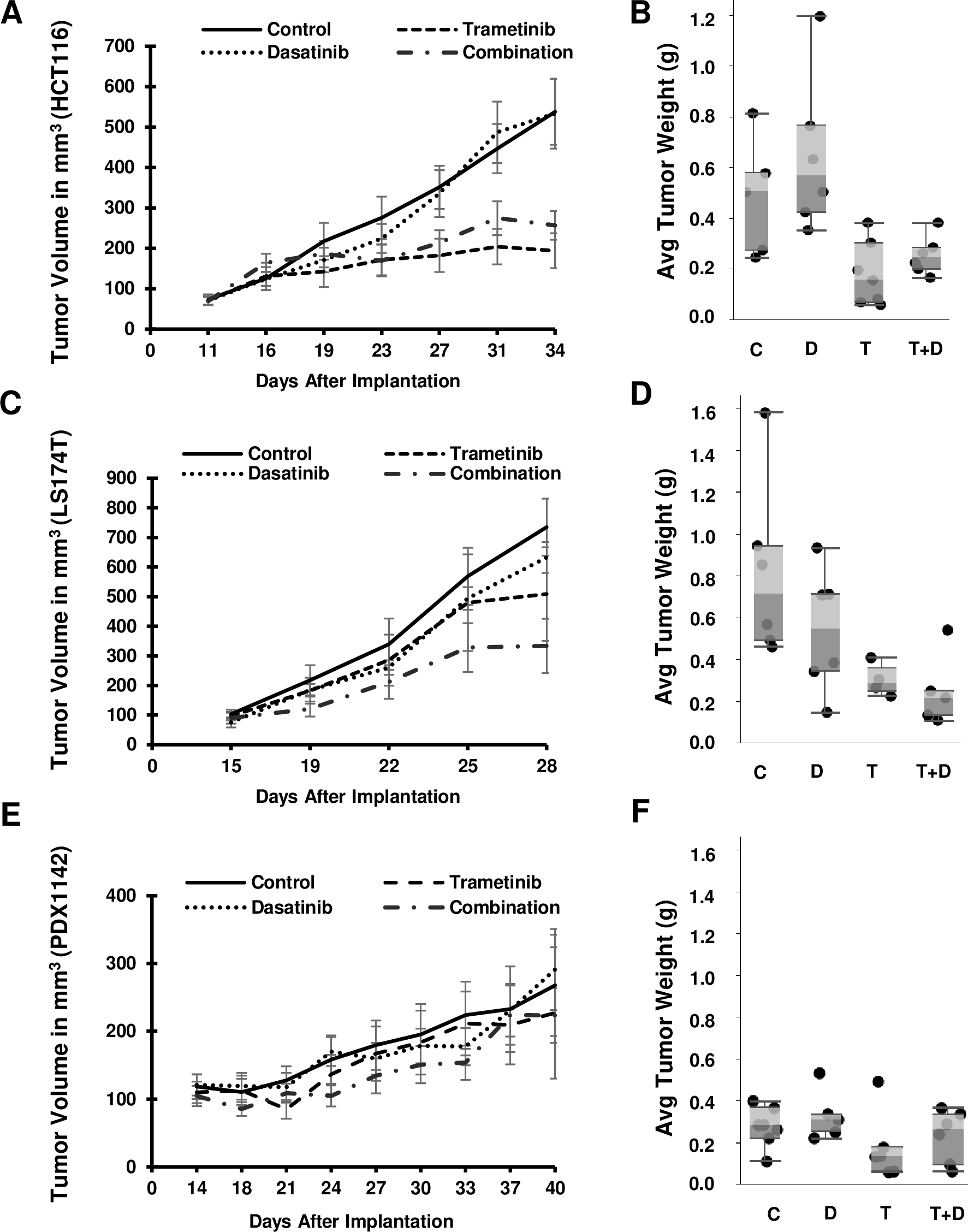

Supplement: S5 Fig — KRAS-mutated CRC cells (HCT116 and LS174T) and CRC PDXs (C1142) were grown subcutaneously in mice, which were given a vehicle (control), dasatinib (10 mg/kg), trametinib (0.2 mg/kg), or dasatinib and trametinib. Tumor volumes were measured on the days shown on the x-axes (tumors implanted on day 0). A, C, and E, graphs of tumor growth. B, D, and F, average weights of residual tumors at the end of the experiments. Note: no significant differences in tumor volume or tumor weight between the combination therapy and trametinib groups were observed. (TIF) [file pone.0281063.s006.tif]

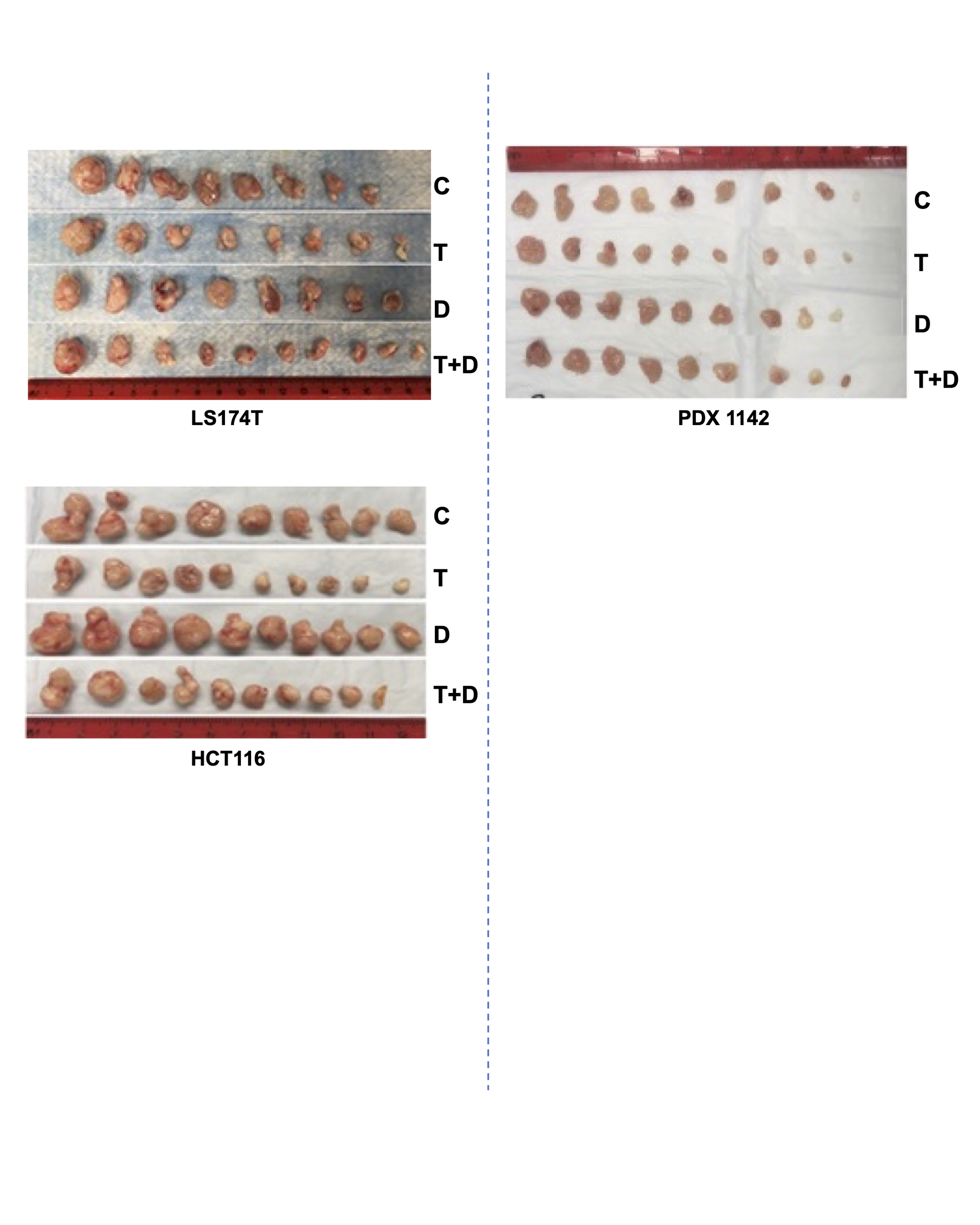

Supplement: S6 Fig — KRAS-mutated CRC cells (LS174T, and HCT116) and CRC PDX (C1142) were grown subcutaneously in mice, which were given a vehicle (C), trametinib (T), dasatinib (D), or trametinib and dasatinib (T+D). Photographs of tumors harvested at the end of the experiments are shown. The cell line and PDX names are shown at the bottom of each panel. Note: all experiments were initiated with 10 mice per group. Some experimental animals died or were euthanized for causes unrelated to treatment during the course of the studies. (TIF) [file pone.0281063.s007.tif]
