# Supplementary material for: Combining MEK and SRC inhibitors for treatment of colorectal cancer demonstrate increased efficacy in vitro but not in vivo
Source: PLoS One. 2023 Mar 23;18(3):e0281063. doi: 10.1371/journal.pone.0281063 (PMC10035898; doi:10.1371/journal.pone.0281063)
Supplement: S1 Raw images — (PDF) [file pone.0281063.s008.pdf]

Figure 3 HCT116 and SW620 cell lines

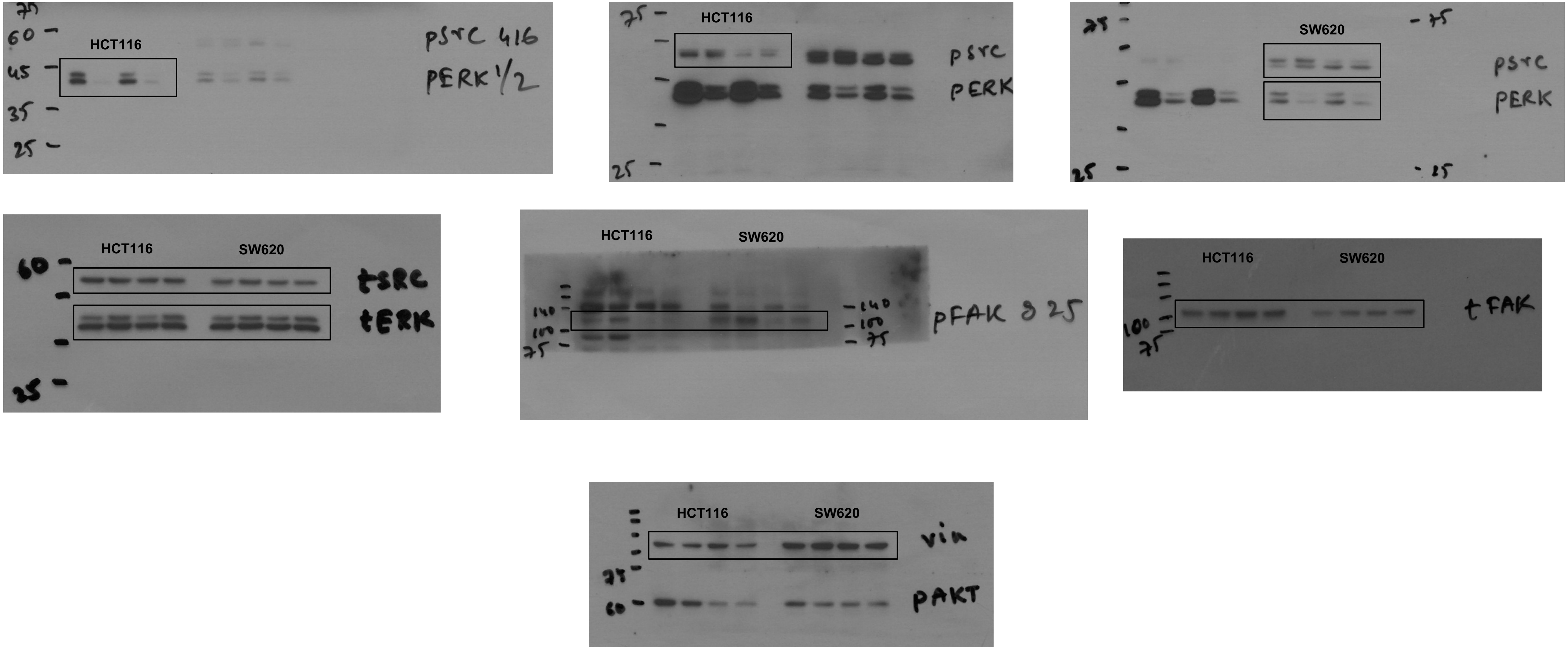

Figure 3 SW480 cell line

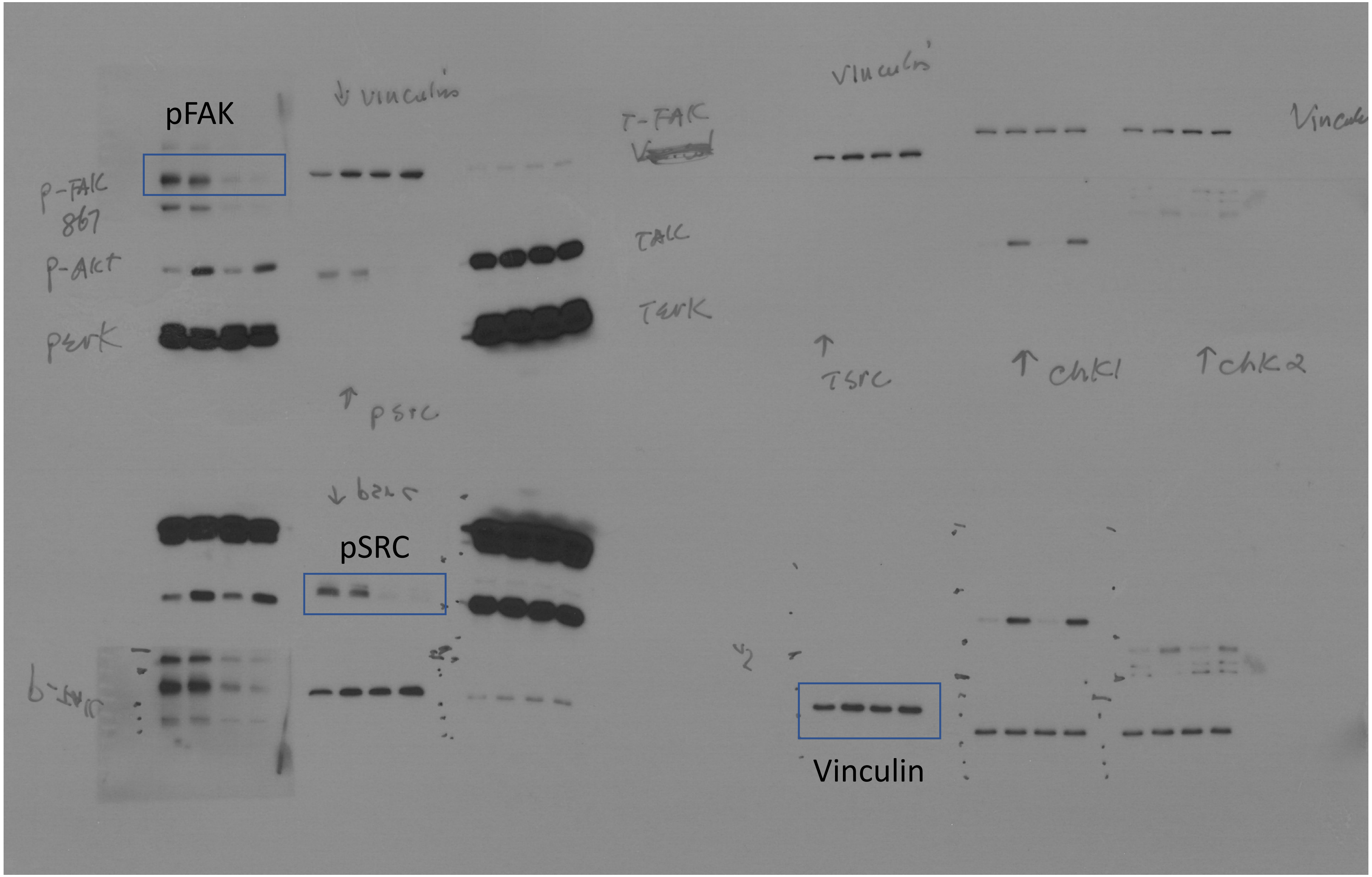

Figure 3 SW480 cell line

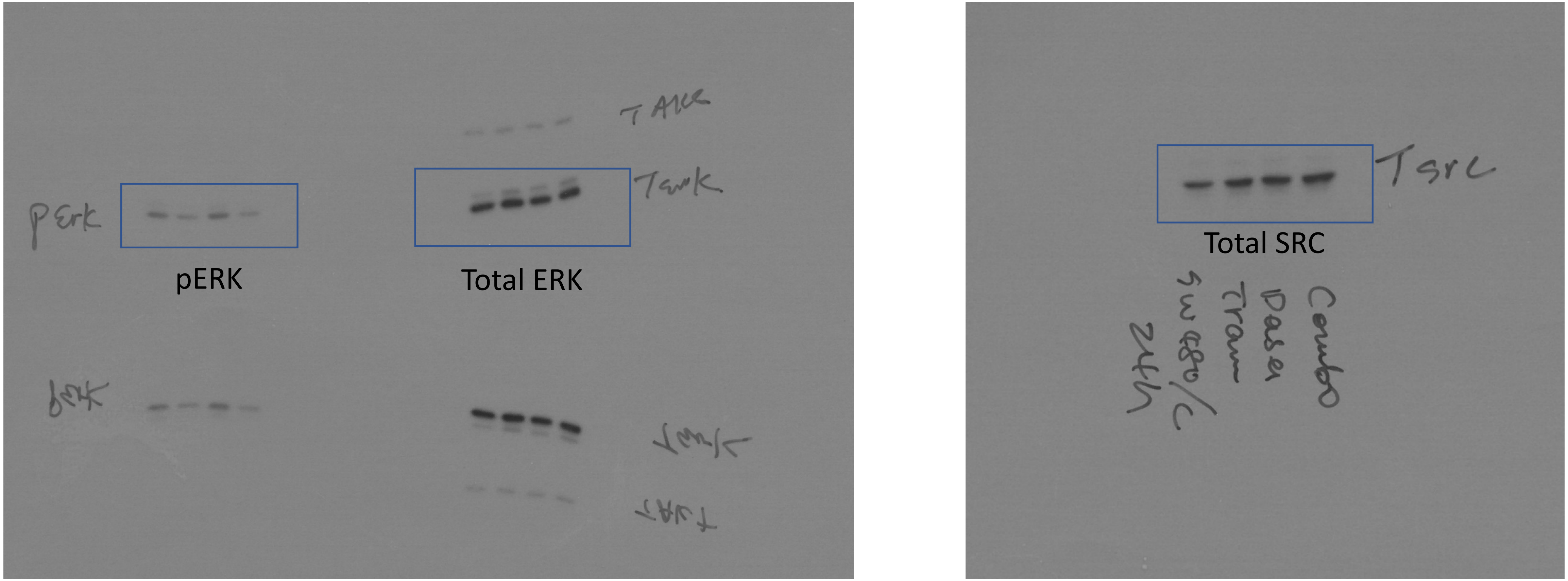

Figure 3 SW480 cell line

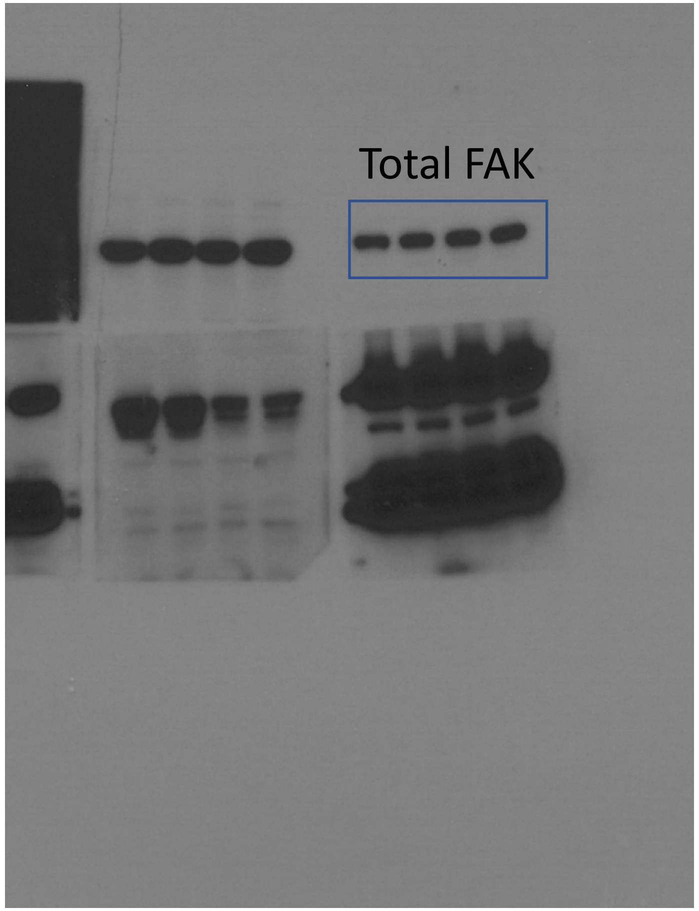

Figure 4

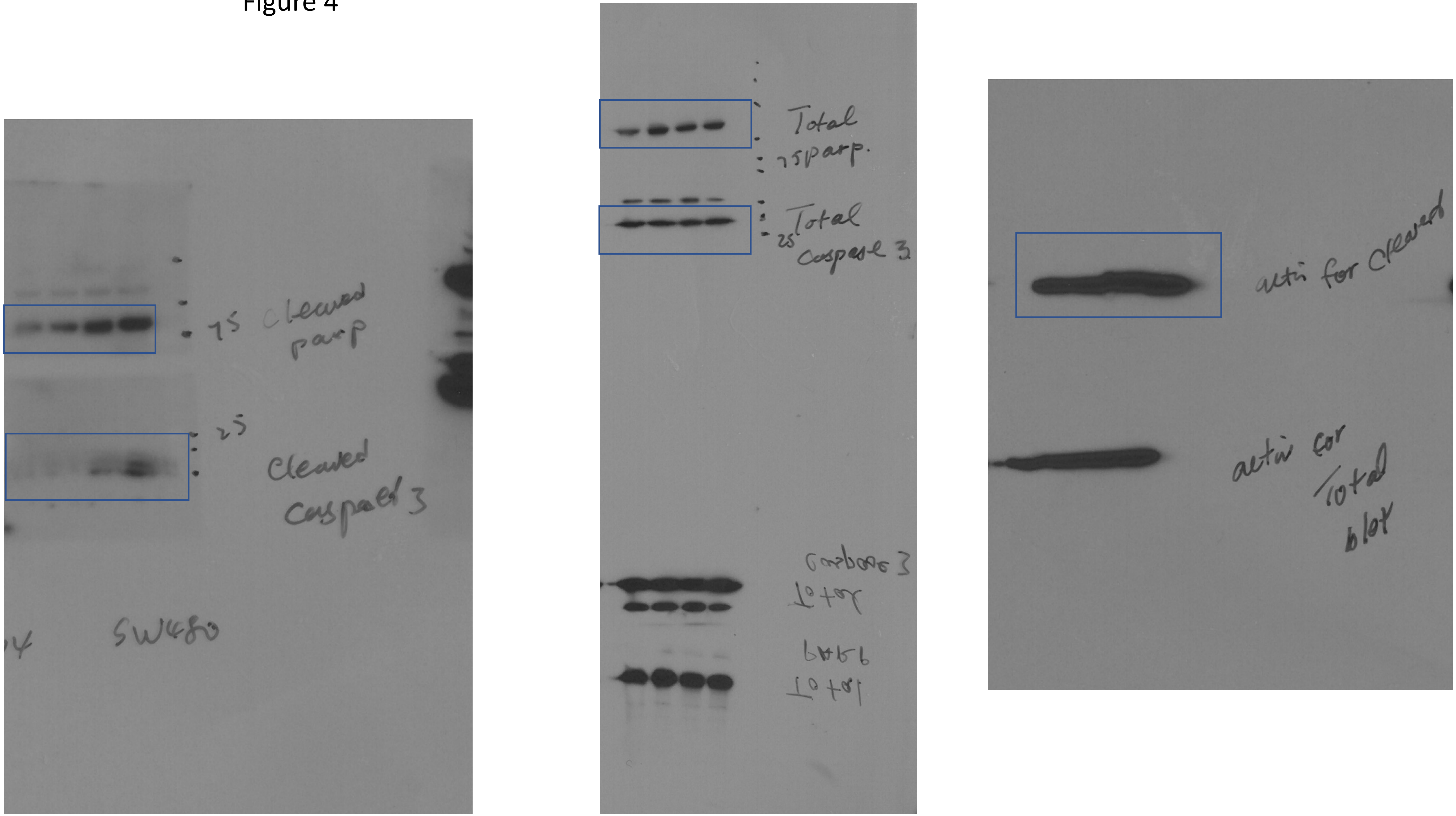
75 cleaved parp
25
cleaved caspase 3
SW480
Total 75 parp
Total 25 caspase 3
actin for cleaved
actin for total blot

Figure 4

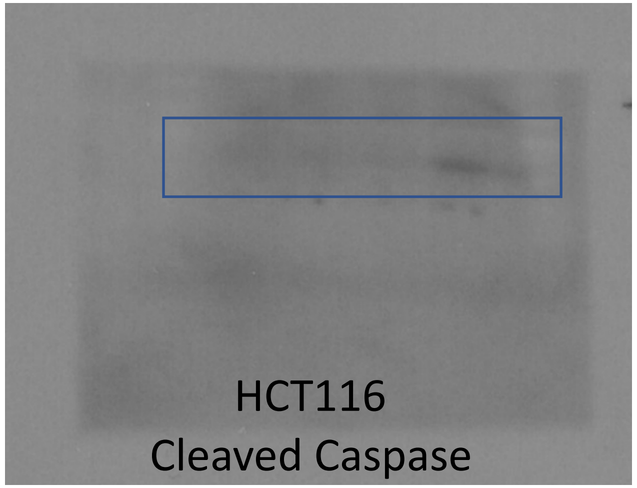
HCT116
Cleaved Caspase

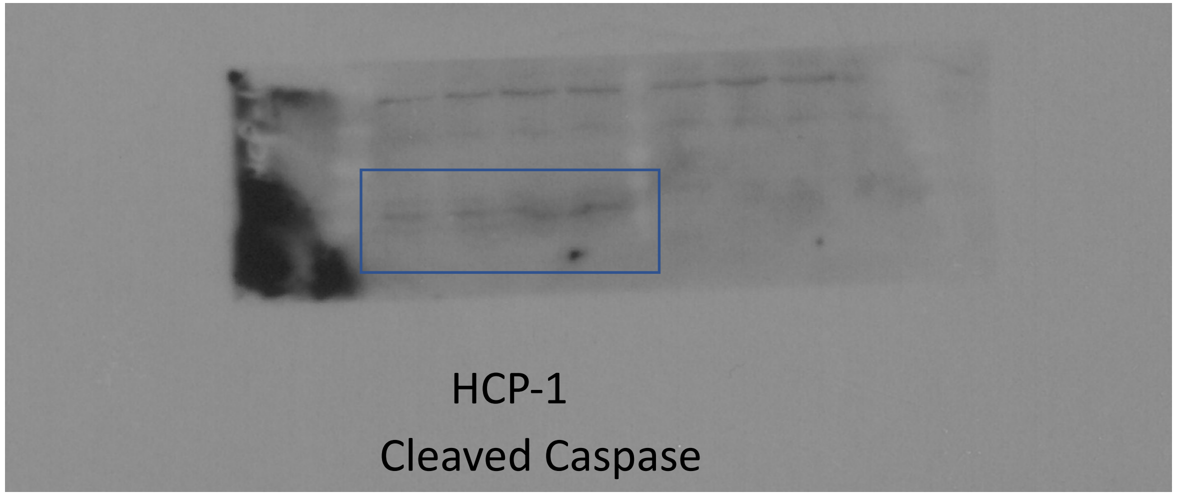
HCP-1
Cleaved Caspase

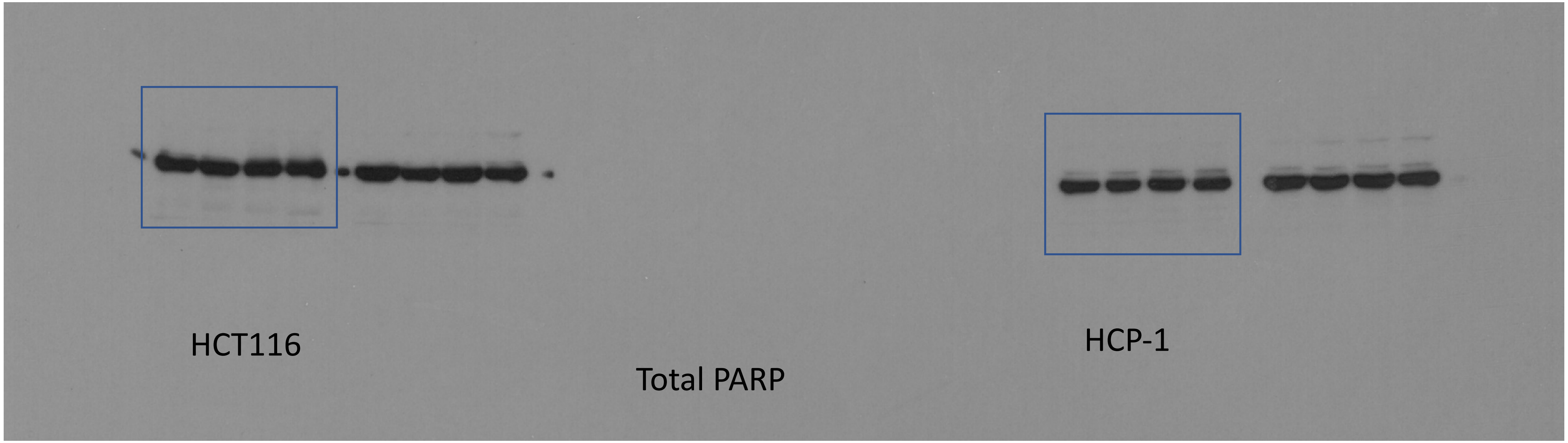
HCT116
Total PARP
HCP-1

Figure 4

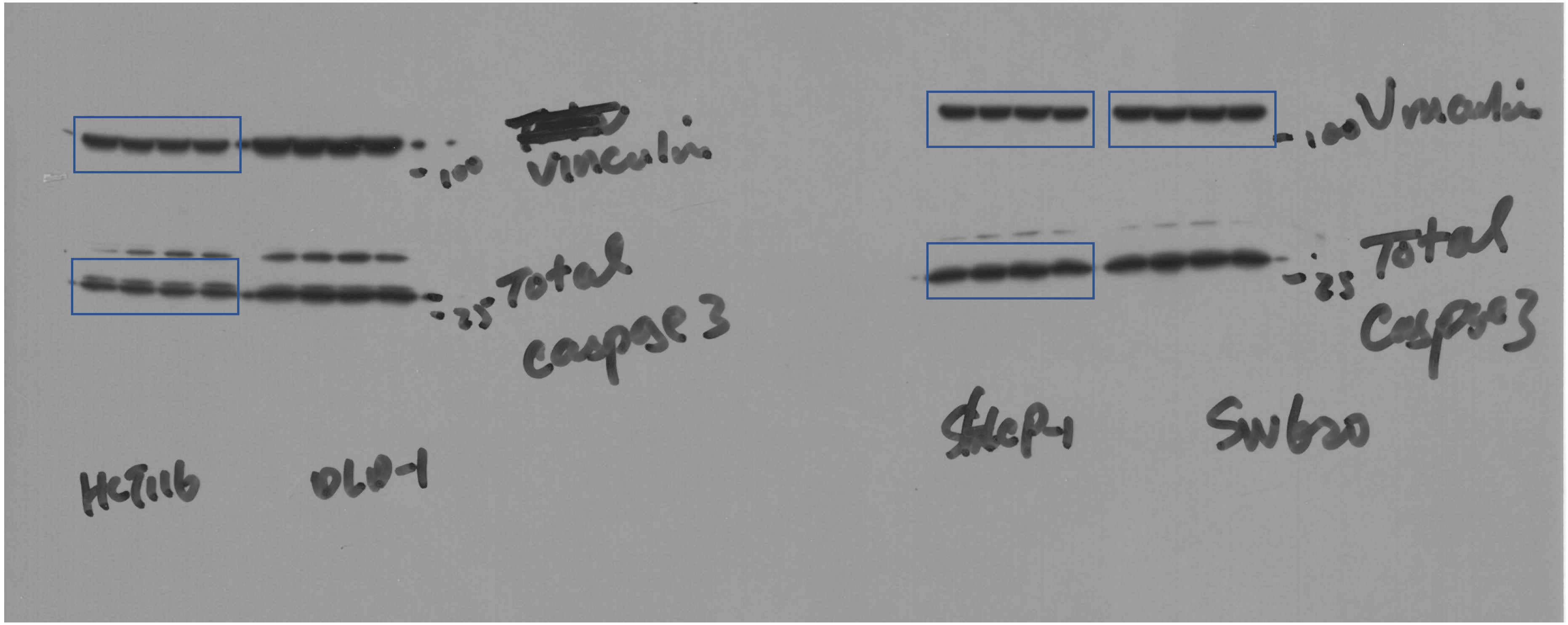

Figure 4

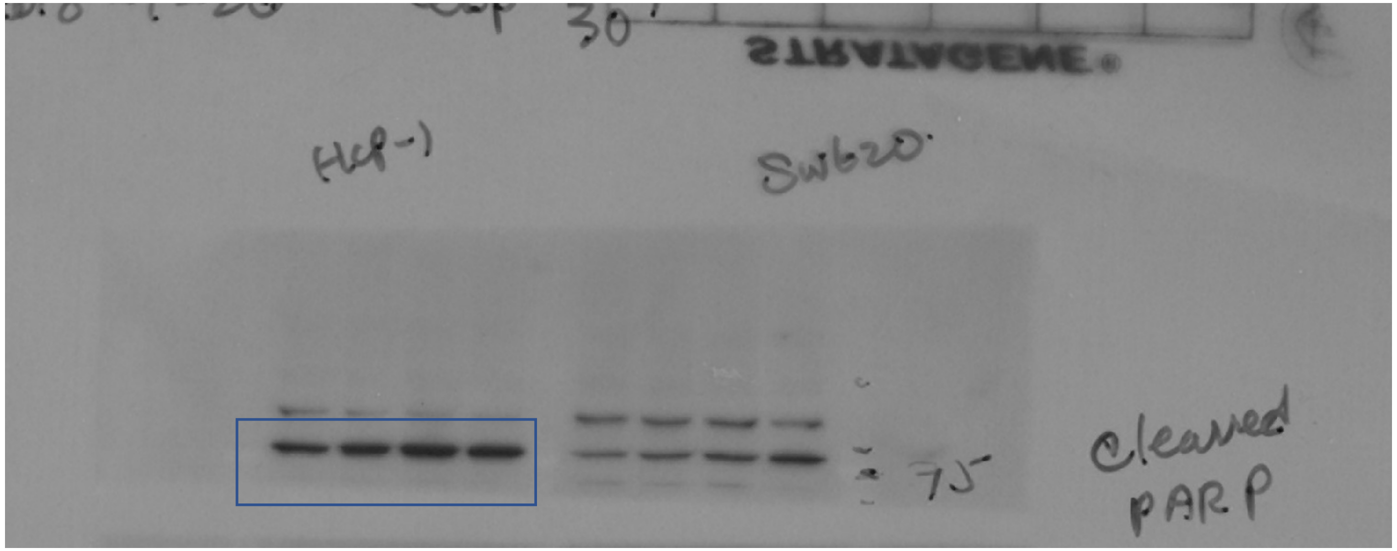

Figure 4

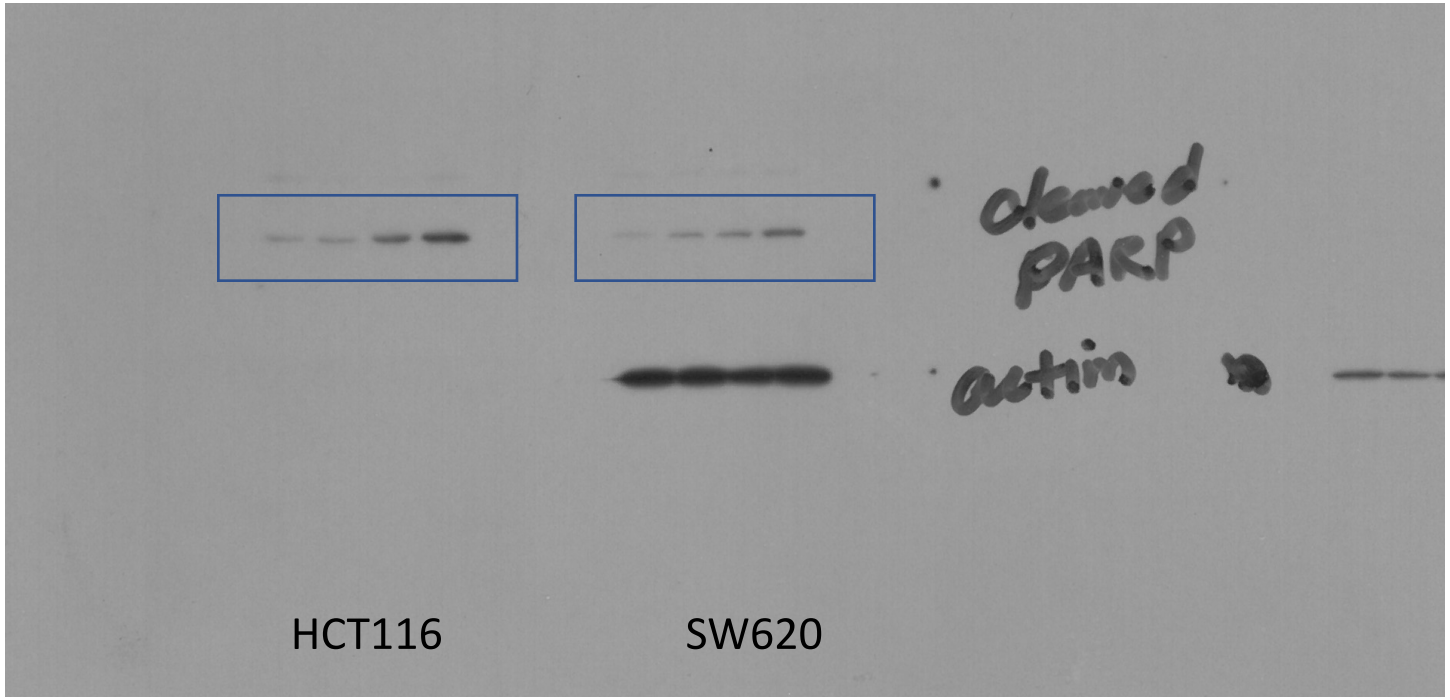

Figure 4

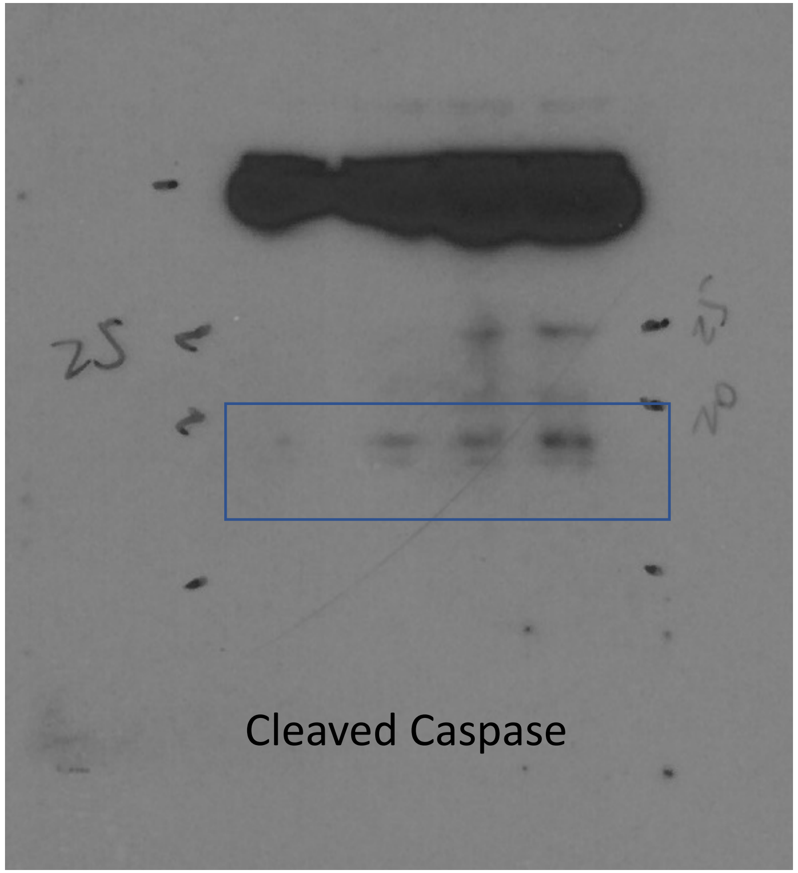
25
Cleaved Caspase

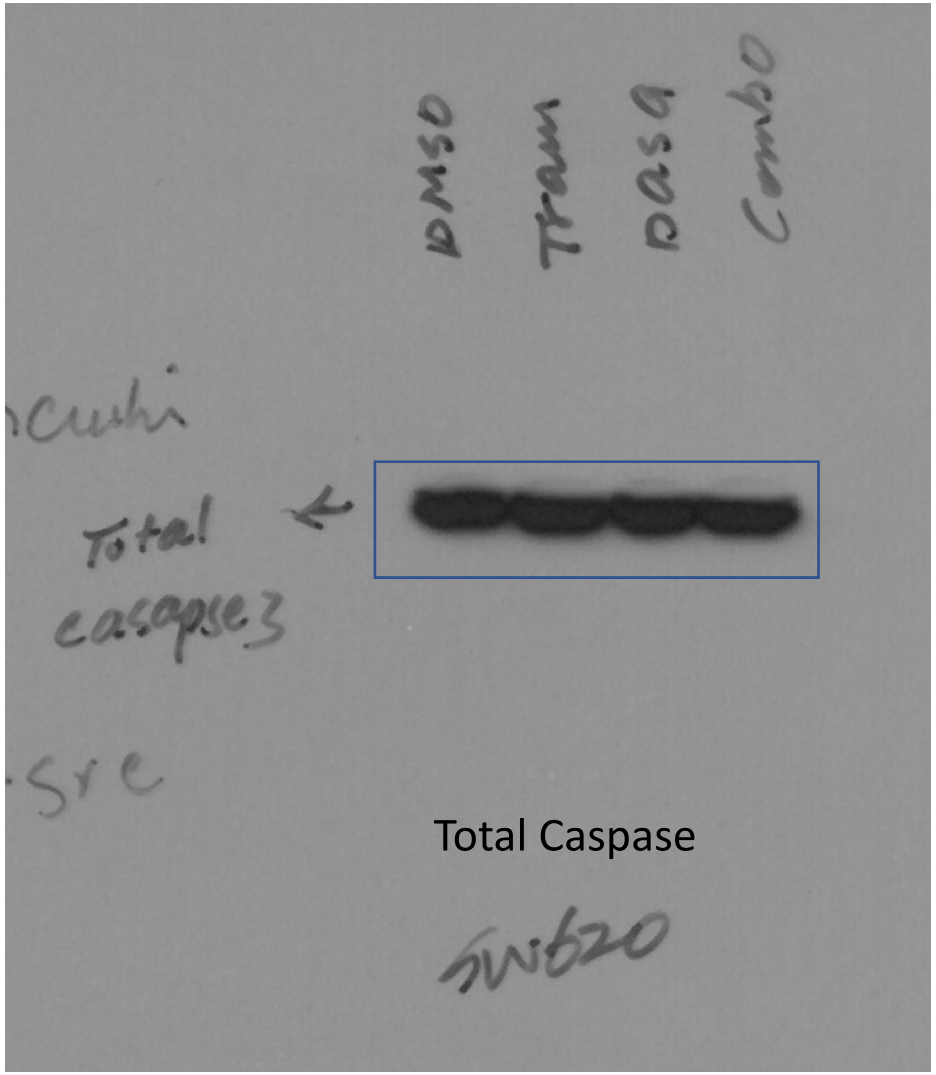
DMSO
Tram
Dasa
Combo
Total
casapse3
Total Caspase
SW620

Figure 5

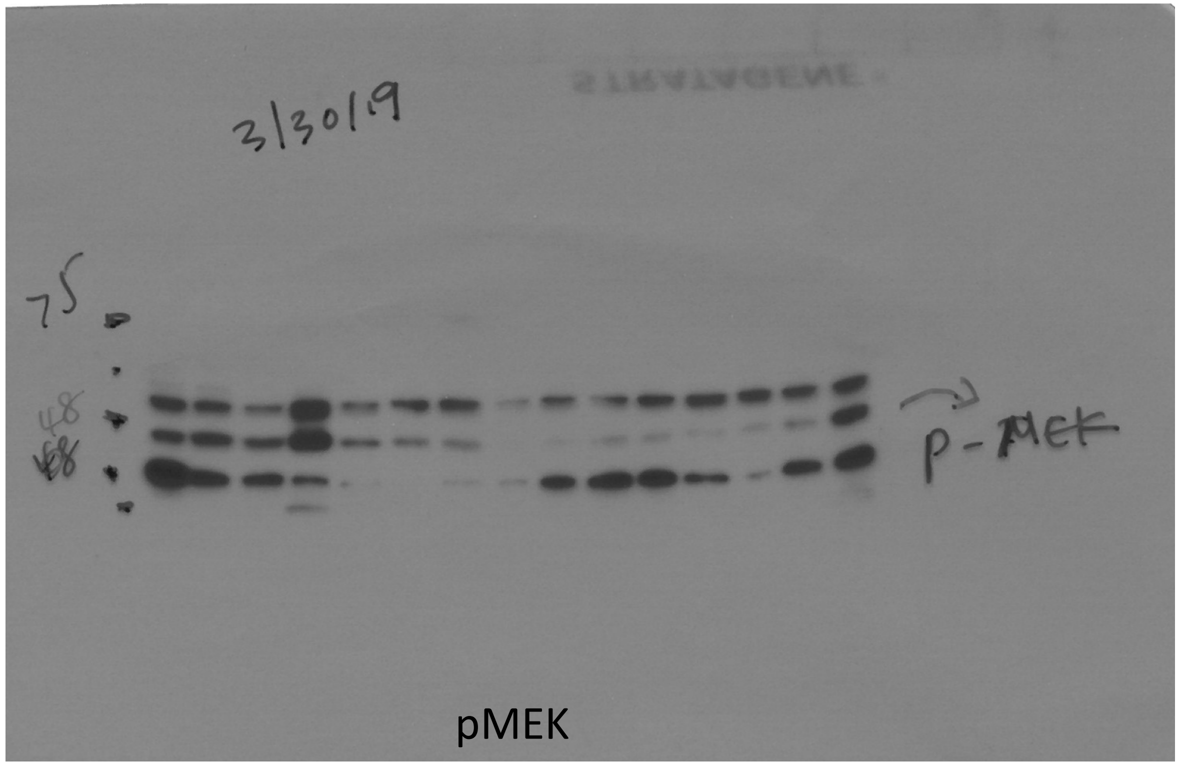

pMEK

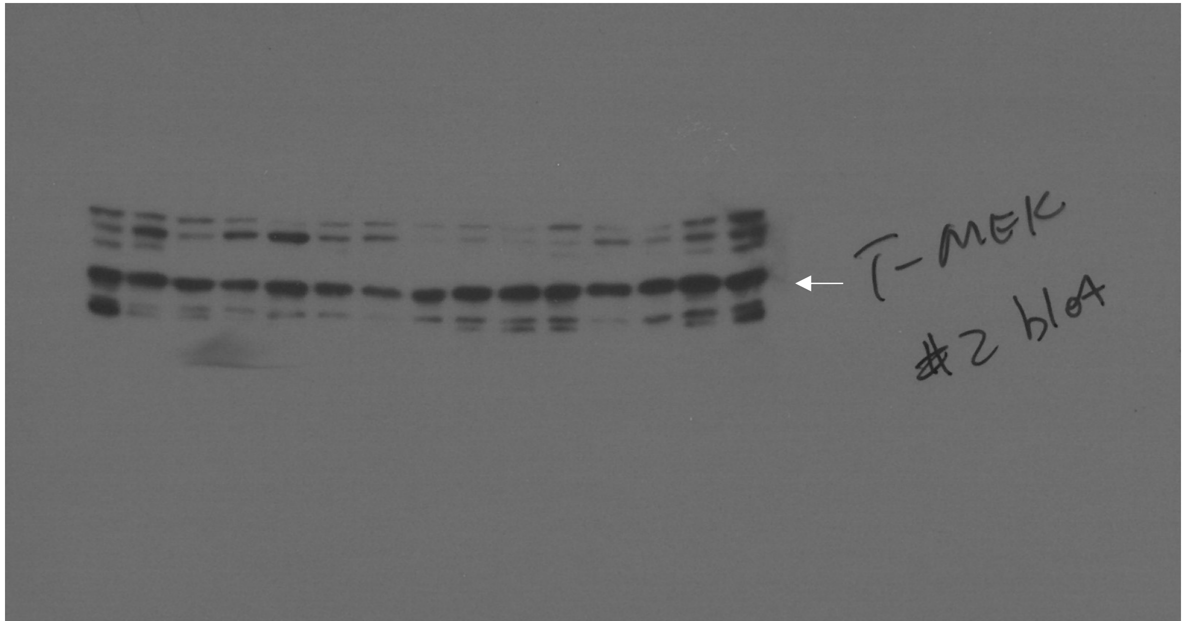

Total MEK

Figure 5

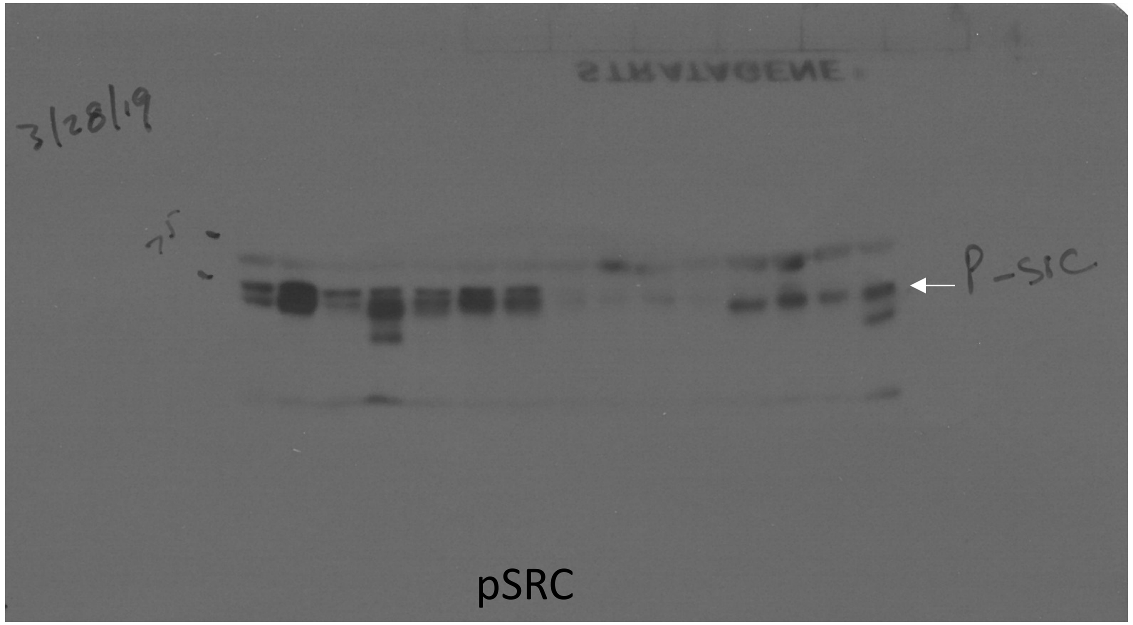

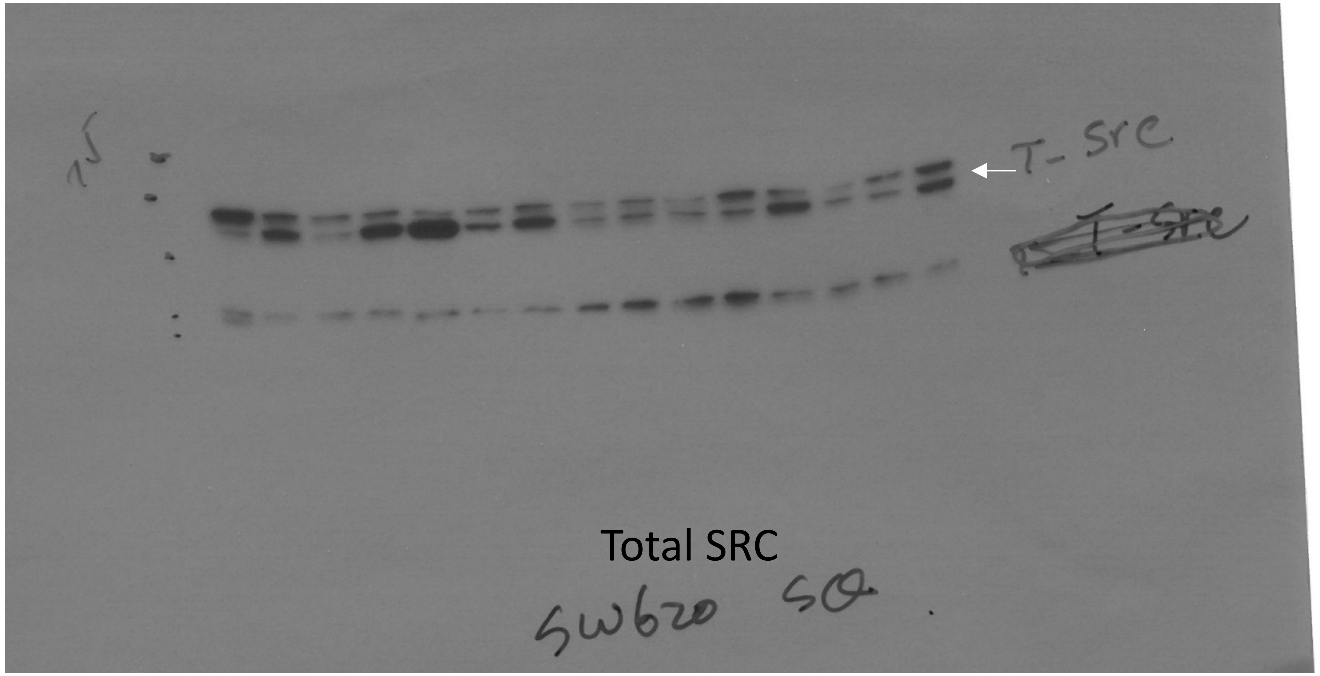

Figure 5

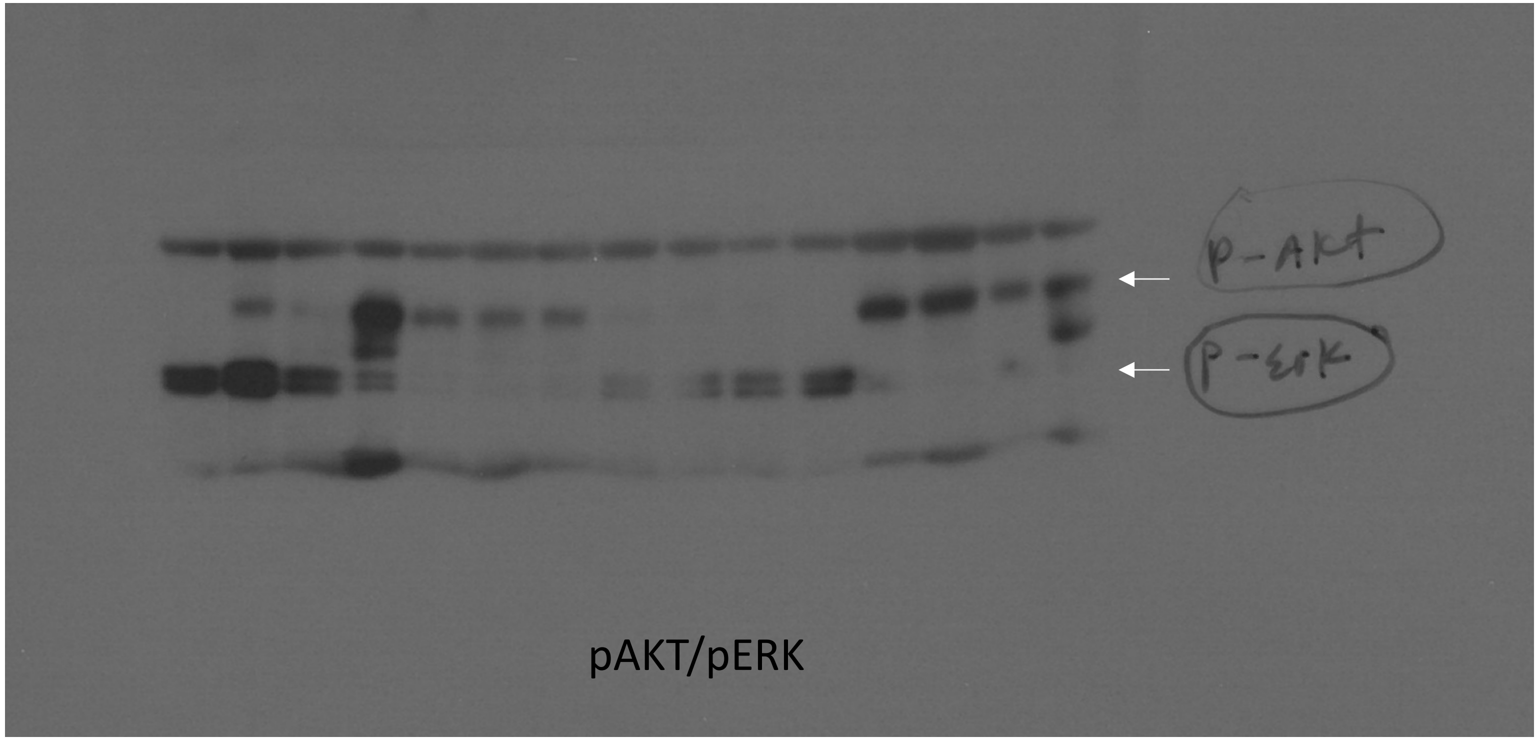

pAKT/pERK

Figure 5

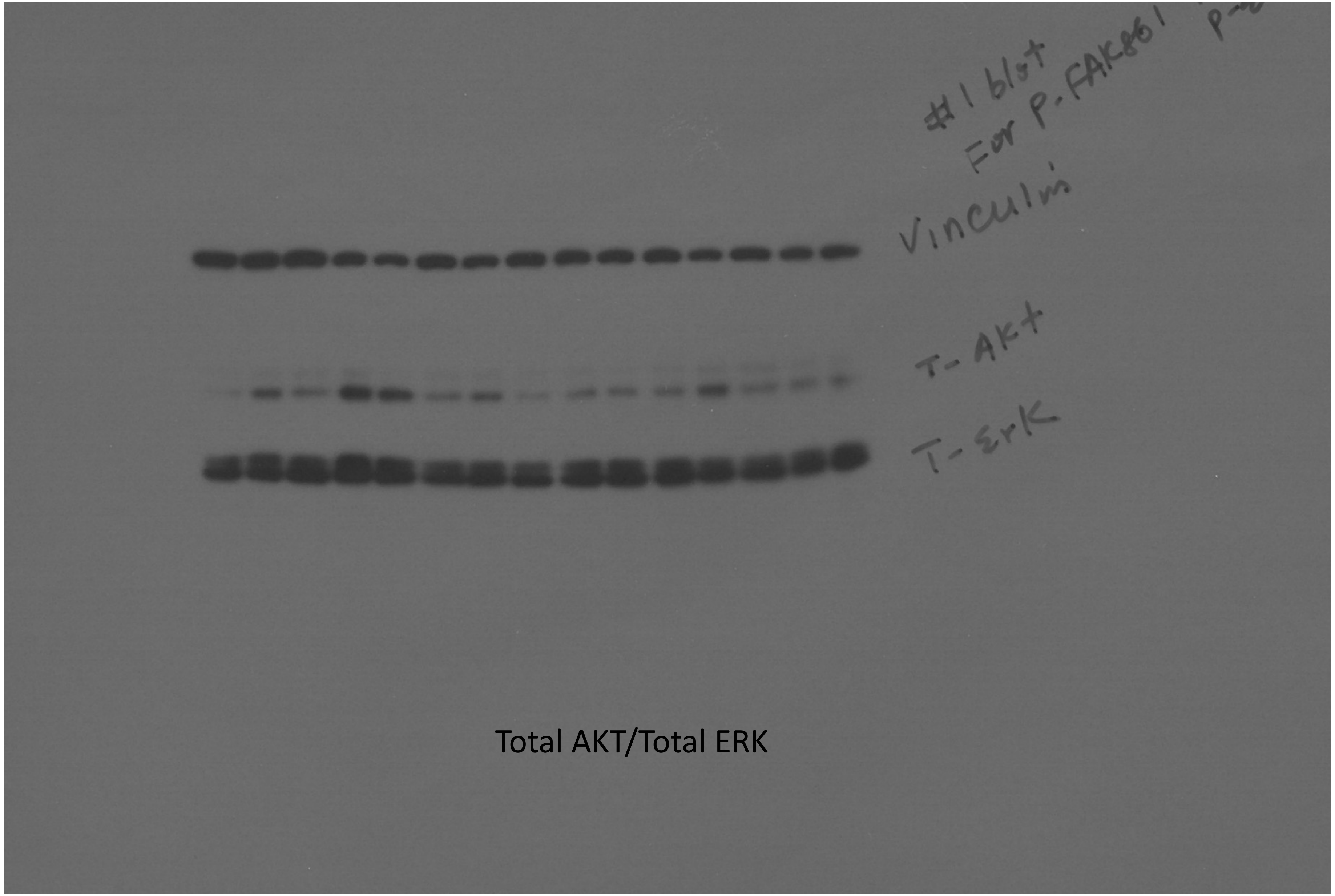

Total AKT/Total ERK
